# Incorporation of Robust NIR‐II Fluorescence Brightness and Photothermal Performance in a Single Large *π*‐Conjugated Molecule for Phototheranostics

**DOI:** 10.1002/advs.202204695

**Published:** 2022-12-01

**Authors:** Yuanyuan Li, Yufu Tang, Wenbo Hu, Zhen Wang, Xi Li, Xiaomei Lu, Shufen Chen, Wei Huang, Quli Fan

**Affiliations:** ^1^ State Key Laboratory for Organic Electronics and Information Displays & Institute of Advanced Materials (IAM) Jiangsu Key Laboratory for Biosensors Nanjing University of Posts & Telecommunications Nanjing 210023 China; ^2^ Key Laboratory of Flexible Electronics (KLOFE) & Institute of Advanced Materials (IAM) Nanjing Tech University 30 South Puzhu Road Nanjing 211800 P. R. China; ^3^ Shaanxi Institute of Flexible Electronics (SIFE) Northwestern Polytechnical University (NPU) Xi'an 710072 China

**Keywords:** flexible side groups, large *π*‐conjugated molecules, photothermal performance, rigid molecular skeletons, second near‐infrared fluorescent brightness

## Abstract

Second near‐infrared (NIR‐II, 1000–1700 nm) window fluorescence imaging‐guided photothermal therapy probes are promising for precise cancer phototheranostics. However, most of the currently reported probes do not demonstrate high NIR‐II fluorescent brightness (molar absorption coefficient (*ε*) × quantum yield (QY)) and photothermal performance (*ε* × photothermal conversion efficiency (PCE)) in a single molecule. Herein, a versatile strategy to solve this challenge is reported by fabricating a large *π*‐conjugated molecule (BNDI‐Me) with a rigid molecular skeleton and flexible side groups. The proposed BNDI‐Me nanoprobe boosts the *ε* and simultaneously optimizes its QY and PCE. Therefore, high NIR‐II fluorescent brightness (*ε* × QY = 2296 m ^−1^ cm^−1^) and strong photothermal performance (*ε* × PCE = 82 000) are successfully incorporated in a single small molecule, and, to the best of knowledge, either of these two parameters is better than the best currently available fluorescent or photothermal probes. Thus, superior NIR‐II imaging effect in vivo and high photothermal tumor inhibition rate (81.2%) at low systemic injection doses are obtained. The work provides further insights into the relationship of photophysical mechanisms and structures, and presents promising molecular design guidelines for the integration of more efficient multiple theranostic functions in a single molecule.

## Graphical abstract

Robust second near‐infrared (NIR‐II) fluorescent brightness and photothermal performance are incorporated in a single small molecule with a rigid molecular skeleton and flexible side groups for improved NIR‐II imaging‐guided photothermal therapy. The work provides further insights into the relationship of photophysical mechanisms and structures, and presents promising molecular design guidelines to integrate multiple theranostic functions in a single molecule.

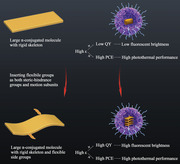

## 1 Introduction

The integration of remarkably high‐resolution near‐infrared‐II (NIR‐II, 1.0–1.7 µm) fluorescence imaging and spatiotemporally controllable heat generation in a single probe, particularly biocompatible and easily repeatable organic small molecules, holds great potential for non‐invasive and precise phototheranostics.^[^ ^1^ ^]^ An ideal small molecule should simultaneously maximize the NIR‐II fluorescence brightness (molar absorption coefficient (*ε*) × quantum yield (QY)) and photothermal performance (*ε* × photothermal conversion efficiency (PCE)). However, this is extremely challenging because the photophysical mechanisms of *ε* (molecular structure of ground state), QY (radiative decay of excited state), and PCE (nonradiative decay of excited state) are often competitive and interrelated in a single molecule.^[^ ^1^, ^2^ ^]^

The current NIR‐II fluorophore design strategy focuses on creating a strong intramolecular charge transfer (ICT) molecular skeleton by incorporating electron donor (D) and acceptor (A) units.^[^ ^3^ ^]^ Generally, D/A subunits linked by covalent single bonds natively prefer flexible intramolecular rotations,^[^ ^4^ ^]^ which not only favor a poor orbital overlap of *π*‐systems, resulting in low *ε*, but also drive the flexible skeleton into a twisted ICT state in strongly polar aqueous bioenvironments to promote the nonradiative decay of the excited state, thereby resulting in a relatively low QY and high PCE.^[^ ^2^, ^4^, ^5^ ^]^ Such materials mainly include polymethine cyanines and D−A−D fluorophores.^[^ ^3^, ^6^ ^]^ Cyanines composed of two heterocycles as D and A typically exhibit appreciable *ε* (≈10^5^ m ^−1^ cm^−1^) in organic solutions; however, attenuated *ε* (≈10^4^ m ^−1^ cm^−1^)^[^ ^7^ ^]^ and low QY (<0.5%)^[^ ^3e^ ^]^ in water limit their practical application. In particular, their low photostability precludes photothermal therapy (PTT) with prolonged exposure to high‐power lasers.^[^ ^3e^ ^]^ In contrast, D−A−D fluorophores usually exhibit higher photostability for lower *ε* (≈10^3^–10^4^ m ^−1^ cm^−1^).^[^ ^2b^ ^]^ In addition, low QY (<1%) and high PCE in water are their common characteristics.^[^ ^2^, ^6^ ^]^ Recent efforts to boost the optical performance of flexible ICT skeleton materials have focused solely on improving any one among *ε*, QY, and PCE,^[^ ^1^, ^2^, ^4^ ^]^ almost at the expense of others owing to their competition and interrelation. For example, the current popular strategy to improve QY involves the incorporation of D with steric rotation barriers into the molecular skeleton to reduce flexible intramolecular rotations.^[^ ^2b^ ^]^ This could raise the QY from 1% to more than 10%, while concomitantly decreasing *ε* from ≈10^4^ to ≈10^3^ m ^−1^ cm^−1^. Thus, the fluorescence brightness is only marginally improved. Another study achieved a PCE of more than 80% by enhancing molecule motion, but the QY (<0.1%) was significantly low.^[^ ^6f^ ^]^ Therefore, an innovative molecular skeleton design strategy that incorporates robust fluorescence brightness and photothermal performance in a single molecule is highly desired.

An unexplored yet possible approach to attain NIR‐II emission involves the implementation of pure *π*‐conjugation (no strong ICT effect) to create a rigid large *π*‐conjugated molecular skeleton. This molecular structure has promising advantages. First, it exhibits a significantly high photostability.^[^ ^8^ ^]^ Second, unlike flexible ICT skeletons, rigid and conjugated skeletons can ensure a rich orbital overlap of *π*‐systems to obtain high *ε*; some reported such first near‐infrared window (NIR‐I, 700–900 nm) emission molecules are as high as 10^8^ m ^−1^ cm^−1^.^[^ ^8^, ^9^ ^]^ Meanwhile, without obvious ICT effects and flexible rotations of subunits in intramolecular skeletons, the aforementioned molecular structure favors radiative decay to ensure a high QY on a molecular level. Although promising, currently reported large *π*‐conjugated molecules, such as naphthalenediimide (NDI) and perylene diimide, only emit NIR‐I;^[^ ^8^ ^]^ those emitting NIR‐II are generally limited by the de novo design and synthesis. Moreover, rigid large *π*‐conjugated skeletons tend to form a highly planar molecule, leading to severe aggregation‐caused quenching (ACQ) in aggregate state in water,^[^ ^8^ ^]^ which results in a significantly low QY and high PCE. Thus, maintaining high *ε* and optimizing the QY and PCE of rigid large *π*‐conjugated molecules in water to incorporate high NIR‐II fluorescence brightness and strong photothermal effect in a single molecule are promising but difficult tasks.

**Scheme 1:**
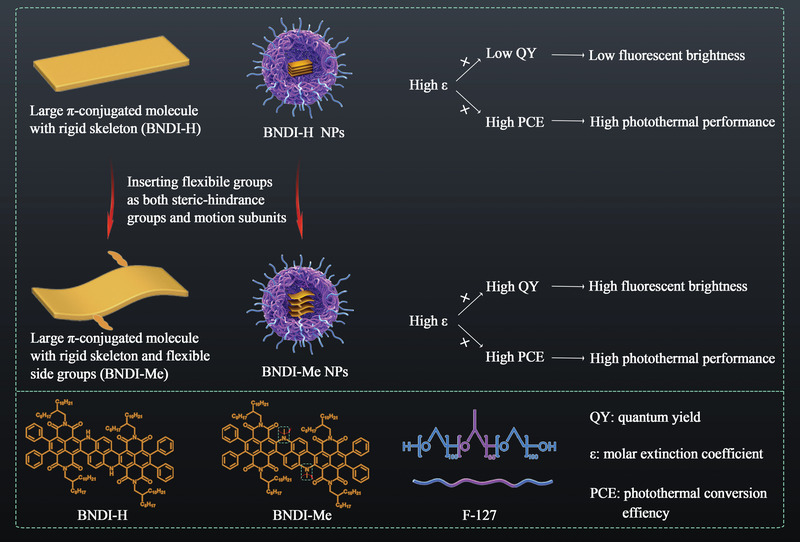
Schematic of the molecular design strategy to incorporate high NIR‐II fluorescence brightness and strong photothermal performance in a single molecule.

We herein report a versatile strategy to incorporate high NIR‐II fluorescence brightness and photothermal performance in a single molecule by fabricating a large *π*‐conjugated small molecule (BNDI‐Me) with a rigid molecular skeleton and flexible side groups (**Scheme** **1**). The rigid and conjugated skeleton of BNDI‐Me ensured a high photostability and a rich orbital overlap of *π*‐systems, resulting in a high *ε* (1.64 × 10^5^ m ^−1^ cm ^−1^) at 859 nm in water. Moreover, BNDI‐Me implemented NIR‐II emission through large *π*‐conjugation rather than a strong ICT effect. Two flexible side groups (methyl groups) in BNDI‐Me acted not only as sterically resistance groups to generate nonplanar molecular skeletons and weaken the ACQ effect to improve QY in water, but also as motion units to enhance PCE, simultaneously obtaining appreciable QY (≈1.4%) and PCE (50%) in water. Thus, BNDI‐Me nanoprobes (NPs) can incorporate high NIR‐II fluorescence brightness (*ε* × QY = 2296) and strong photothermal performance (*ε* × PCE = 82 000) in a single molecule, and, to the best of our knowledge, either of these two parameters is better than the best currently available fluorescent or photothermal probes (Table S1, Supporting Information). As a proof‐of‐concept, BNDI‐Me NPs exhibited superior NIR‐II imaging quality in vivo with a high photothermal tumor inhibition rate of 81.2% for 4T1 tumor.

## 2 Results and Discussion

### 2.1 Molecular Design and Synthesis

The molecular design of BNDI‐H and BNDI‐Me is illustrated in Scheme 1. NDI was selected because it has a rigid large *π*‐conjugated molecular skeleton without a strong ICT effect.^[^ ^8^ ^]^ The new large *π*‐conjugated molecule (BNDI‐H) had a novo design; it was synthesized by broadening the conjugate skeleton of NDI dye. BNDI‐H emissions appear in an NIR‐II window owing to its large *π*‐conjugation rather than a strong ICT effect. Its molecular skeleton is rigid and conjugated without a strong ICT effect, which favors the radiative decay of the excited state to ensure high QY on a molecular level. However, the rigid and conjugated molecular skeletons tend to form planar molecules, leading to strong intermolecular *π*‐*π* interaction in the aggregation state,^[^ ^9^ ^]^ which results in severe ACQ with significantly low QY and high PCE in water. To overcome this issue, two flexible side groups (methyl groups) were inserted in the middle of BNDI‐H as steric‐hindrance groups and motion subunits.^[^ ^10^ ^]^ As steric‐hindrance groups, these methyl groups twist the plane of BNDI‐H to increase the intermolecular space and reduce aggregation, thus boosting NIR‐II fluorescent QY but decreasing the PCE. Moreover, as motion subunits, the motion of these methyl groups assists heat generation, which offsets the decrease in heat due to reduced aggregation. Therefore, two flexible side groups optimize the QY and PCE of BNDI‐Me NPs to obtain appreciable QY and PCE simultaneously. In addition, the rigid and conjugated molecular skeleton of BNDI‐Me ensures a rich orbital overlap of the *π*‐system, resulting in a high *ε*. Conclusively, BNDI‐Me obtains high *ε* and simultaneously optimizes the QY and PCE in water, which incorporates high NIR‐II fluorescence brightness and strong photothermal effect in a single molecule.

The synthetic routes of BNDI‐H and BNDI‐Me are presented in Scheme S1, Supporting Information. The organic small molecule, BrNDI‐H, was synthesized by a nucleophilic aromatic substitution reaction between 4,5,9,10‐tetrabromo‐2,7‐bis(2‐octyldodecyl)benzo[lmn] Phenanthroline‐1,3,6,8(2H,7H)‐tetraone (4Br‐NDI) and naphthalene‐1,5‐diamine. Next, tributylphenylstannane and BrNDI‐H were used in a Stille coupling reaction to form BNDI‐H. BNDI‐Me was further synthesized based on a methylation reaction with BNDI‐H. The chemical structures of BrNDI‐H, BNDI‐H, and BNDI‐Me were characterized by the NMR spectra and mass analysis (Figures S1–S9, Supporting Information). BNDI‐H and BNDI‐Me demonstrated good solubility in common organic solvents such as tetrahydrofuran, chloroform, and toluene.

**Figure 1:**
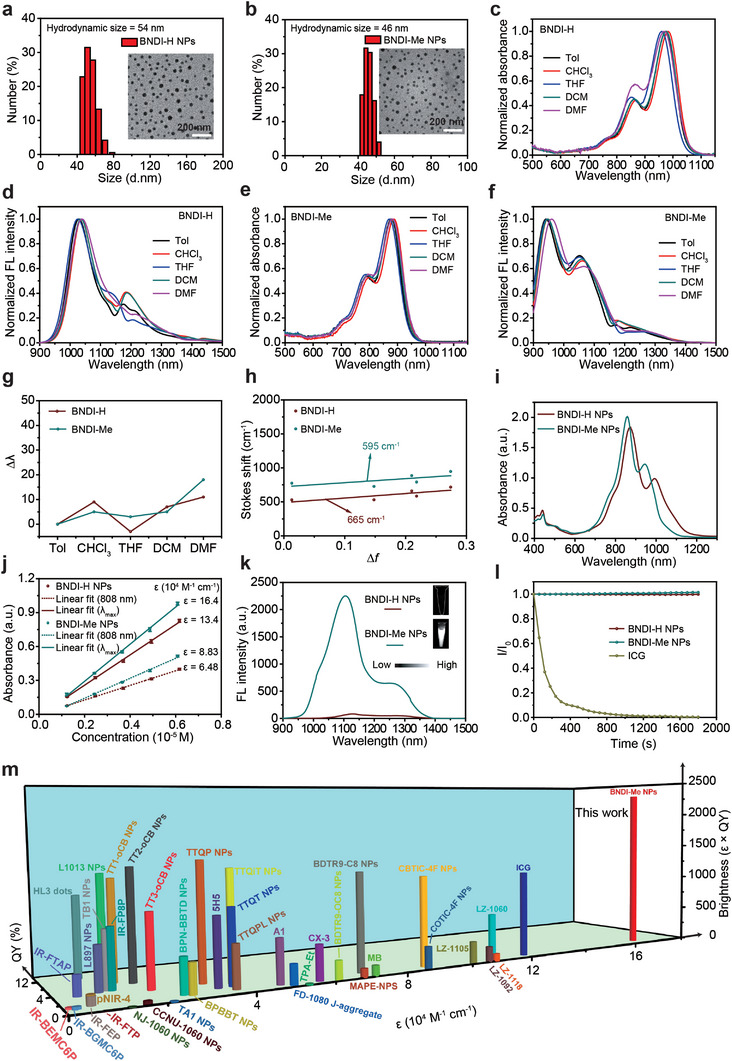
Characterization of BNDI‐H and BNDI‐Me and their NPs. DLS and TEM images (inset) of a) BNDI‐H NPs and b) BNDI‐Me NPs. Normalized absorption and fluorescence spectra of c,d) BNDI‐H and e,f) BNDI‐Me in different polar solvents. g) Maximum emission wavelength increment (∆*λ*) in BNDI‐H and BNDI‐Me in different polar solvents. The solvent polarity parameter Δ*f*: Toluene (Tol) < Chloroform (CHCl_3_) < tetrahydrofuran (THF) < dichloromethane (DCM) < dimethylformamide (DMF). ∆*λ* is defined as the emission peak of both molecules in the solvent (Tol, CHCl_3_, THF, DCM, and DMF) minus the emission peak in toluene. h) Linear fitting of Stokes shifts versus solvent polarity parameter (Δ*f*) in different solvents. i) Absorption spectra of BNDI‐H NPs and BNDI‐Me NPs with the same molar concentration. j) Plot of absorption values at 808 nm and maximum absorption peak versus concentration of BNDI‐H NPs and BNDI‐Me NPs. The bars are mean ± s.d. (*n* = 3). k) Fluorescence spectra of BNDI‐H NPs and BNDI‐Me NPs at the same molar concentration (excitation: 808 nm). The inset shows NIR‐II fluorescence images of BNDI‐H NPs and BNDI‐Me NPs with the same concentration under LP 980 nm filter. l) Photostability of BNDI‐H NPs, BNDI‐Me NPs, and indocyanine green (ICG) in aqueous solution upon continuous 808 nm (1 W cm^−2^) exposure for 30 min. m) NIR‐II fluorescence brightness of previously reported organic fluorophores and BNDI‐Me NPs. Detailed data are listed in Table S1 and Figure S27, Supporting Information.

To achieve good water dispersibility and biocompatibility, hydrophobic BNDI‐H and BNDI‐Me were co‐precipitated with the amphiphilic polymer F‐127 to fabricate BNDI‐H NPs and BNDI‐Me NPs. The average hydrodynamic diameters of BNDI‐H NPs and BNDI‐Me NPs were found to be ≈54 and 46 nm, respectively, by dynamic light scattering (DLS) measurement (**Figure** **1**a,b). The transmission electron microscopy (TEM) images of BNDI‐H NPs and BNDI‐Me NPs exhibited good homogeneous spherical morphologies (Figure 1a,b inset). It should be noted that the size of BNDI‐H NPs and BNDI‐Me NPs remained nearly unchanged for 5 days, exhibiting great structural stability (Figure S10, Supporting Information).

### 2.2 Optical Properties

To verify BNDI‐H and BNDI‐Me without a strong ICT effect, the classical solvatochromic effect test was first conducted for BNDI‐H and BNDI‐Me in different solvent polarities.^[^ ^5^, ^11^ ^]^ As shown in Figure 1c–f, BNDI‐H and BNDI‐Me exhibited the expected NIR‐I absorption peak and NIR‐II emission. As the solvent polarity increased from toluene to dimethylformamide, the fluorescence peak of BNDI‐H (Figure 1d) and BNDI‐Me (Figure 1f) only changed slightly. More importantly, their fluorescence peaks demonstrated random changes instead of progressive redshifts when the solvent polarity was increased (Figure 1g). This contradicts the phenomenon that the fluorescence peak of strong ICT molecules gradually shifts into long wavelengths when the solvent polarity is increased.^[^ ^5^, ^11^ ^]^ Furthermore, the relationship between the Stokes shift (*ν̃*_abs_–*ν̃*_em_) and solvent polarity parameter (∆*f*) was fitted to evaluate the bathochromic shift feature according to the Lippert–Mataga equation.^[^ ^5a^ ^]^ As shown in Figure 1h, the linear relationship of ∆*f* and Stokes shift exhibits a slope of 665 and 595 cm^−1^ for BNDI‐H and BNDI‐Me, respectively, which is considerably less than the value of the reported strong ICT molecules.^[^ ^5a^ ^]^ Such spectrum features indicate that both BNDI‐H and BNDI‐Me do not exhibit a strong ICT effect. Therefore, BNDI‐H and BNDI‐Me attain NIR‐II emission based on large *π*‐conjugation rather than a strong ICT effect. This may overcome the native limitation of strong ICT molecules. For example, the instinctive drawback of a strong ICT molecule is that its low‐energy excited state can be seriously quenched by strongly polar water molecules in biological environments, thus favoring high nonradiative decay with a significantly low QY.^[^ ^2^, ^5^ ^]^ However, the optical behavior of a large *π*‐conjugated molecule is almost insensitive to environmental polarity variation; in addition, its excited state tends to undergo radiative decay, which is beneficial for improving the QY.

Next, the optical properties of BNDI‐H NPs and BNDI‐Me NPs were studied. As shown in Figure 1i, both NPs displayed broad absorption, ranging from 600 to 1200 nm. BNDI‐H NPs and BNDI‐Me NPs exhibited major absorption peaks at ≈873 and 859 nm with shoulder peaks at 991 and 944 nm, respectively. The values of *ε* at their major absorption peaks were determined to be 1.34 × 10^5^ for BNDI‐H NPs and 1.64 × 10^5^ m ^−1^∙cm^−1^ for BNDI‐Me NPs (Figure 1j and Figure S11, Supporting Information), which are one or two orders of magnitude higher than those of strong ICT molecules (≈10^3^–10^4^ m ^−1^∙cm^−1^) and cyanines (≈10^4^ m ^−1^∙cm^−1^) in water,^[^ ^7^ ^]^ thereby reflecting the advantage of large polycyclic *π*‐conjugated molecules in terms of light absorbing abilities. In addition, although their absorption at 808 nm (commonly used excitation wavelength for theranostics) was significantly weaker than their major peak absorption (Figure 1i), *ε* was still as high as 6.48 × 10^4^ and 8.83 × 10^4^ m ^−1^∙cm^−1^ for BNDI‐H NPs and BNDI‐Me NPs, respectively (Figure 1j). The emission peaks of BNDI‐H NPs and BNDI‐Me NPs were observed around 1128 and 1104 nm, respectively, in the aqueous solution under 808 nm laser excitation (Figure 1k). The fluorescent intensity of BNDI‐H NPs was evidently weaker than that of BNDI‐Me NPs with the same molar concentration, which agreed with their NIR‐II fluorescence images (Figure 1k inset).

Furthermore, their NIR‐II fluorescence QY was evaluated. As expected, the NIR‐II fluorescence QY of BNDI‐Me NPs (≈1.4%) was ≈23 times higher than that of BNDI‐H NPs (≈0.06%) in the aqueous solution, with IR‐26 (QY of IR‐26 = 0.5%)^[^ ^12^ ^]^ as a reference system (Figure S12, Supporting Information). The QY of BNDI‐Me NPs was evidently higher than that of some reported organic NIR‐II fluorescence materials (Table S1 and Figure S27, Supporting Information). The NIR‐II fluorescence brightness, the main criterion for imaging performance evaluation in fluorescent molecules, was assessed based on *ε* × QY values.^[^ ^2b^ ^]^ Accordingly, the *ε* × QY values of BNDI‐H NPs and BNDI‐Me NPs at major absorption peak wavelengths were calculated to be 80.4 and 2296, respectively. The *ε* × QY value of BNDI‐Me NPs (2296) was ≈28.6 times higher than that of BNDI‐H NPs (80.4), and evidently higher than that of most of previously reported NIR‐II fluorophores with high fluorescence brightness (Figure 1m; Table S1 and Figure S27, Supporting Information). Compared to severe photobleaching in Food and Drug Administration‐approved indocyanine green (ICG) (Figure 1l), the fluorescent intensity of BNDI‐H NPs and BNDI‐Me NPs demonstrated negligible attenuation under continuous 808 nm (1 W cm^−2^) irradiation for 30 min, thereby exhibiting excellent photostability. Meanwhile, BNDI‐Me NPs exhibited excellent photostability in serum (Figure S13, Supporting Information). Moreover, the fluorescence intensity of BNDI‐Me NPs was also unaffected by the temperature ranging from 25 to 45 °C, indicating its superior structural stability in the body temperature (Figures S14, Supporting Information). Thus, such high fluorescence brightness, especially in BNDI‐Me NPs, is appropriate for NIR‐II fluorescence imaging in vivo.

### 2.3 Photothermal Properties

**Figure 2:**
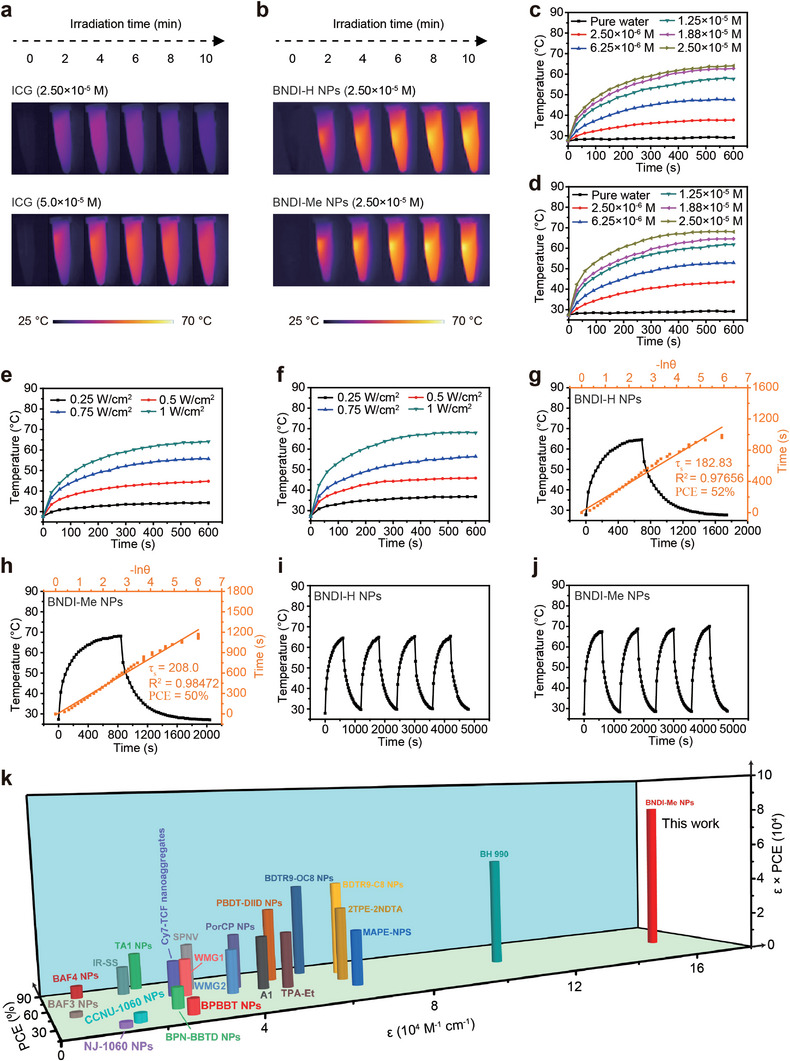
Photothermal properties of BNDI‐H NPs and BNDI‐Me NPs. Photothermal images of a) ICG and b) two NPs under an 808 nm laser irradiation (1 W cm^−2^, 10 min). Concentration‐dependent temperature change curves of c) BNDI‐H NPs and d) BNDI‐Me NPs under irradiation by 808 nm laser (1 W cm^−2^) within 10 min. Laser power density‐dependent temperature change curves of e) BNDI‐H NPs and f) BNDI‐Me NPs at a concentration of 2.50 × 10^−5^ m under irradiation by 808 nm laser. Calculation of photothermal conversion efficiencies of g) BNDI‐H NPs and h) BNDI‐Me NPs (808 nm, 1 W cm^−2^). Black line: Temperature variation curve of BNDI‐H NPs and BNDI‐Me NPs under 808 nm laser irradiation, followed by the natural cooling process. Yellow line: Linear time data versus −Ln *θ* during the cooling period. Temperature variation curves of i) BNDI‐H NPs and j) BNDI‐Me NPs over four cycles under on/off irradiation by 808 nm laser. k) Photothermal performance (*ε* × PCE) of BNDI‐Me NPs and previously reported high‐efficiency organic photothermal agents. Detailed data are listed in Table S1 and Figure S27, Supporting Information.

To study the photothermal properties of both NPs, clinically approved ICG was used as the control. As shown in **Figure** **2**a,b, when both NPs and ICG were present at the same concentration, the solution temperature of ICG was found to be ≈40 °C, which started to decrease after laser irradiation for 4 min owing to its poor photostability (Figure 2a (top) and Figure S15, Supporting Information). Even when the concentration was doubled, the solution temperature of ICG could only reach ≈49 °C at ≈6 min, and then decreased (Figure 2a (down) and Figure S15, Supporting Information). In contrast, the solution temperature of both NPs continuously increased (Figure 2b) and the maximum temperature was obviously higher than that of ICG. This indicates that both NPs are superior to ICG for PTT. Furthermore, the concentration and laser power density‐dependent temperature change curves of BNDI‐H NPs and BNDI‐Me NPs were tested under 808 nm laser irradiation. Under continuous irradiation with an 808 nm laser (1 W cm^−2^, 10 min), all solution temperatures of BNDI‐H NPs and BNDI‐Me NPs (Figure 2c,d, respectively) gradually increased when the probe concentration was increased from 2.50 × 10^−6^ to 2.50 × 10^−5^ m. The maximum temperature of BNDI‐H NPs and BNDI‐Me NPs was found to be ≈64.1 and 68 °C, respectively, at 2.50 × 10^−5^ m. In the control experiment, the temperature was increased by ≈1.8 °C for pure water under 808 nm laser irradiation (Figure 2c,d). Furthermore, temperature changes in BNDI‐H NPs and BNDI‐Me NPs (Figure 2e,f, respectively) at a concentration of 2.50 × 10^−5^ m were studied under different laser power from 0.25 to 1 W cm^−2^. The solution temperature of both NPs rapidly increased with the laser power density. These results unambiguously confirmed that BNDI‐H NPs and BNDI‐Me NPs are efficient photothermal agents. According to a previously reported method,^[^ ^13^ ^]^ the PCE of BNDI‐H NPs and BNDI‐Me NPs was calculated to be 52% and 50%, respectively, under 808 nm laser irradiation (Figure 2g,h, Figure S16, Supporting Information). Four heating/cooling cycles of BNDI‐H NPs and BNDI‐Me NPs indicated their good photothermal ability (Figure 2i,j and Figure S16, Supporting Information). The photothermal performances of both NPs exhibited almost no attenuation, thus confirming their excellent photothermal stability. Furthermore, we studied the absorption spectra change of BNDI‐H NPs (Figure S17a, Supporting Information) and BNDI‐Me NPs (Figure S17b, Supporting Information) through continuous 808 nm (1 W cm^−2^) laser irradiation for 30 min. The absorption spectra and solution color exhibited negligible change. However, the absorption of clinically popular ICG aqueous solution significantly decreased, and the color of the solution shifted from green to pale yellow (Figure S17c, Supporting Information). It further confirmed the photostability of BNDI‐H NPs and BNDI‐Me NPs. Finally, we further evaluated the photothermal performance of both NPs based on *ε* × PCE.^[^ ^14^ ^]^ The value of *ε* × PCE for BNDI‐H NPs and BNDI‐Me NPs at their maximum absorption wavelength was calculated to be 6.97 × 10^4^ and 8.20 × 10^4^, respectively. More importantly, the value of BNDI‐Me NPs was evidently higher than that of previously reported best organic photothermal probes (Figure 2k; Table S1 and Figure S27, Supporting Information), to the best of our knowledge, indicating that BNDI‐Me NPs demonstrate better photothermal performance. Moreover, the excellent photothermal properties and photothermal stability of BNDI‐Me NPs make them a promising candidate for PTT in vivo.

### 2.4 Possible Photophysical Mechanism to Incorporate High Fluorescence Brightness and Photothermal Performance

**Figure 3:**
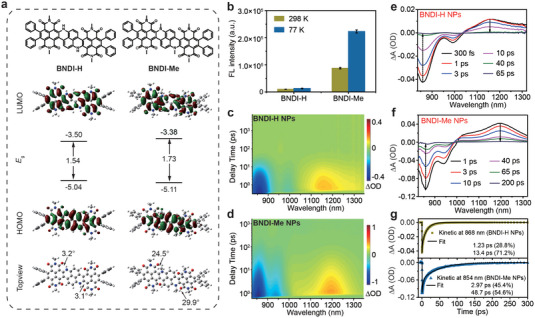
Photophysical mechanism study to incorporate high fluorescence brightness and photothermal performance. a) DFT‐calculated HOMO/LUMO (top view) and geometry (top view) of BNDI‐H and BNDI‐Me (alkyl side groups on NDI are replaced by methyl in the calculations). b) Quantified NIR‐II fluorescence intensity of BNDI‐H and BNDI‐Me in THF solution at 298 and 77 K under 808 nm laser irradiation. Data are plotted as the mean ± SD; *n* = 3. 2D color‐coded maps of fs‐TA spectra of c) BNDI‐H NPs and d) BNDI‐Me NPs under excitation at 800 nm. fs‐TA spectra of e) BNDI‐H NPs and f) BNDI‐Me NPs at selected decay times. g) Kinetic decay curve of GSB at 868 and 854 nm for BNDI‐H NPs and BNDI‐Me NPs, respectively, and fitting lines (solid line).

Different from the traditional strategy of improving the fluorescence brightness at the expense of photothermal performance, the aforementioned experimental data show that BNDI‐Me NPs achieved improved QY while maintaining an appreciable PCE. To demonstrate the possible mechanism, we first performed density functional theory (DFT) calculations on the electronic cloud distributions of BNDI‐H and BNDI‐Me (**Figure** **3**a). As shown in Figure 3a, the highest occupied molecular orbital (HOMO) and lowest unoccupied molecular orbital (LUMO) of BNDI‐H and BNDI‐Me were almost evenly delocalized along the conjugated skeleton, but they were not mainly located on the local units. It also confirmed both BNDI‐H and BNDI‐Me without a strong ICT effect, consistent with the classical solvatochromic effect test (Figure 1c–h). The HOMO, LUMO, and bandgap of BNDI‐H were −5.04, −3.50, and 1.54 eV, respectively. However, BNDI‐Me exhibited a lower HOMO (−5.11 eV), higher LUMO (−3.38 eV), and increased bandgap (1.73 eV), which are characteristic of blue shifts in the absorption spectrum of BNDI‐Me compared to BNDI‐H, consistent with the spectrum data (Figure 1c,e).

To confirm the two flexible methyl groups as steric groups for the formation of nonplanar BNDI‐Me, the optimized ground state geometries of the molecules were further calculated. BNDI‐H exhibited small dihedral angles of 3.2° and 3.1° between the NDI unit and its core (Figure 3a (down)). However, BNDI‐Me demonstrated larger dihedral angles of 24.5° and 29.9°, indicating that BNDI‐Me exhibits nonplanar conformation. Thus, these data indicate that two methyl groups as steric groups caused nonplanar conformation in BNDI‐Me. To further verify that nonplanar conformation indeed reduced the ACQ effect in aggregates, the ACQ effect of BNDI‐H and BNDI‐Me in different water fractions was studied (Figure S18, Supporting Information). BNDI‐H exhibited faster decay than BNDI‐Me when the water fraction was increased, indicating that more severe ACQ was observed in highly planar BNDI‐H in comparison to nonplanar BNDI‐Me. This was consistent with the phenomenon that the QY of BNDI‐Me NPs was ≈23 times higher than that of BNDI‐H NPs (Figure S12, Supporting Information). These data showed that two flexible methyl groups as steric groups to form nonplanar BNDI‐Me could effectively increase the intermolecular spaces in nanoparticles, thus weakening the ACQ effect in comparison to planar BNDI‐H and improving the QY of BNDI‐Me NPs.

It is worth noting that by weakening the molecular ACQ, the molecular PCE can be reduced.^[^ ^8^ ^]^ However, the PCE of BNDI‐Me NPs (50%) did not decrease significantly in comparison to that of BNDI‐H NPs (52%). Previous reports have demonstrated that flexible methyl groups can be used as motion units to improve PCE,^[^ ^10^ ^]^ which may offset a decrease in heat due to reduced aggregation. Thus, we hypothesized that the two methyl groups of BNDI‐Me acted as not only steric groups but also motion units. To verify our hypothesis, the fluorescence of BNDI‐H and BNDI‐Me was first analyzed under different temperatures in THF because low temperatures can restrict intramolecular motion to increase fluorescence.^[^ ^6n^ ^]^ When the temperature was changed from high (298 K) to low (77 K), the fluorescence of BNDI‐Me in THF was enhanced by ≈2.55 times, while that of BNDI‐H was enhanced by only 1.33 times (Figure 3b and Figure S19, Supporting Information). This indicates that the two methyl groups of BNDI‐Me acted as motion units, which successfully verifies our hypothesis that the motion of the methyl groups provided a new nonradiative decay channel to offset the decrease in PCE due to reduced aggregation in comparison to BNDI‐H. Thus, BNDI‐Me NPs also demonstrated a high PCE (50%), which was significantly close to that of BNDI‐H NPs (52%). Conclusively, the two flexible methyl groups acted as steric groups and motion units to optimize the QY and PCE.

To further understand the radiation and non‐radiation features of BNDI‐H NPs and BNDI‐Me NPs, we investigated the excited state dynamics of both NPs through femtosecond transient absorption (fs‐TA) spectrum measurements. According to the steady‐state absorption spectrums (Figure 1i) and after‐ward biotheranostic application, we used an 800 nm pulse laser as excitation laser to study the fs‐TA spectrum of both NPs. Generally, ground state bleaching (GSB) and stimulated emission (SE) generally exhibit negative signals. Excited state absorption (ESA) generally exhibits positive signals. Based on the steady‐state absorption spectrums (Figure 1i), prominent negative signals should be assigned to GSB (Figure 3c,d). Positive signals were attributed to ESA.^[^ ^6^, ^13^ ^]^ To further achieve more details, fs‐TA spectra of the both NPs (Figure S20, Supporting Information and Figure 3e,f) at selected decay times were extracted from Figure 3c,d, respectively. After excitation, GSB and ESA signals showed rapidly rising and reached their maximum values, that is, ≈300 fs for BNDI‐H NPs and 1 ps for BNDI‐Me NPs (Figure S20, Supporting Information). As the delay time increased, both of them gradually decreased, and basically decay ended within 65 ps for BNDI‐H NPs and 200 ps for BNDI‐Me NPs, which excluded the emergence of other new species (Figure 3e,f). GSB usually represents the repopulation of the ground state by nonradiative decay process of the excited state. Thus, the GSB decay processes at 868 nm for BNDI‐H NPs and 854 nm for BNDI‐Me NPs were fitted in Figure 3g and are listed in Table S2, Supporting Information. Both BNDI‐H NPs and BNDI‐Me NPs deactivated through two different nonradiative channels. The corresponding relative amplitudes (A_1_ or A_2_) could be used to evaluate the fraction of the excited population that decays through the different channels. The nonradiative decay channel for BNDI‐H NPs shows considerably short lifetimes of 1.23 ps (*τ*_1_) and 13.4 ps (*τ*_2_) of the excited population with corresponding amplitudes of 28.8% (A_1_) and 71.2% (A_2_) (Figure 3g and Table S2, Supporting Information). For BNDI‐Me NPs, the nonradiative decay channel dissipates considerably short lifetimes of 2.97 ps (*τ*_1_) and 48.7 ps (*τ*_2_) of the excited population with corresponding amplitudes of 45.4% (A_1_) and 54.6% (A_2_). Notedly, the average nonradiative decay lifetime of BNDI‐H NPs (9.90 ps) is obviously shorter than that of BNDI‐Me NPs (27.94 ps) (Table S2, Supporting Information). Compared with BNDI‐Me NPs, the faster nonradiative decay of BNDI‐H NPs causes their weaker fluorescence.

### 2.5 NIR‐II Fluorescence Imaging In Vivo

**Figure 4:**
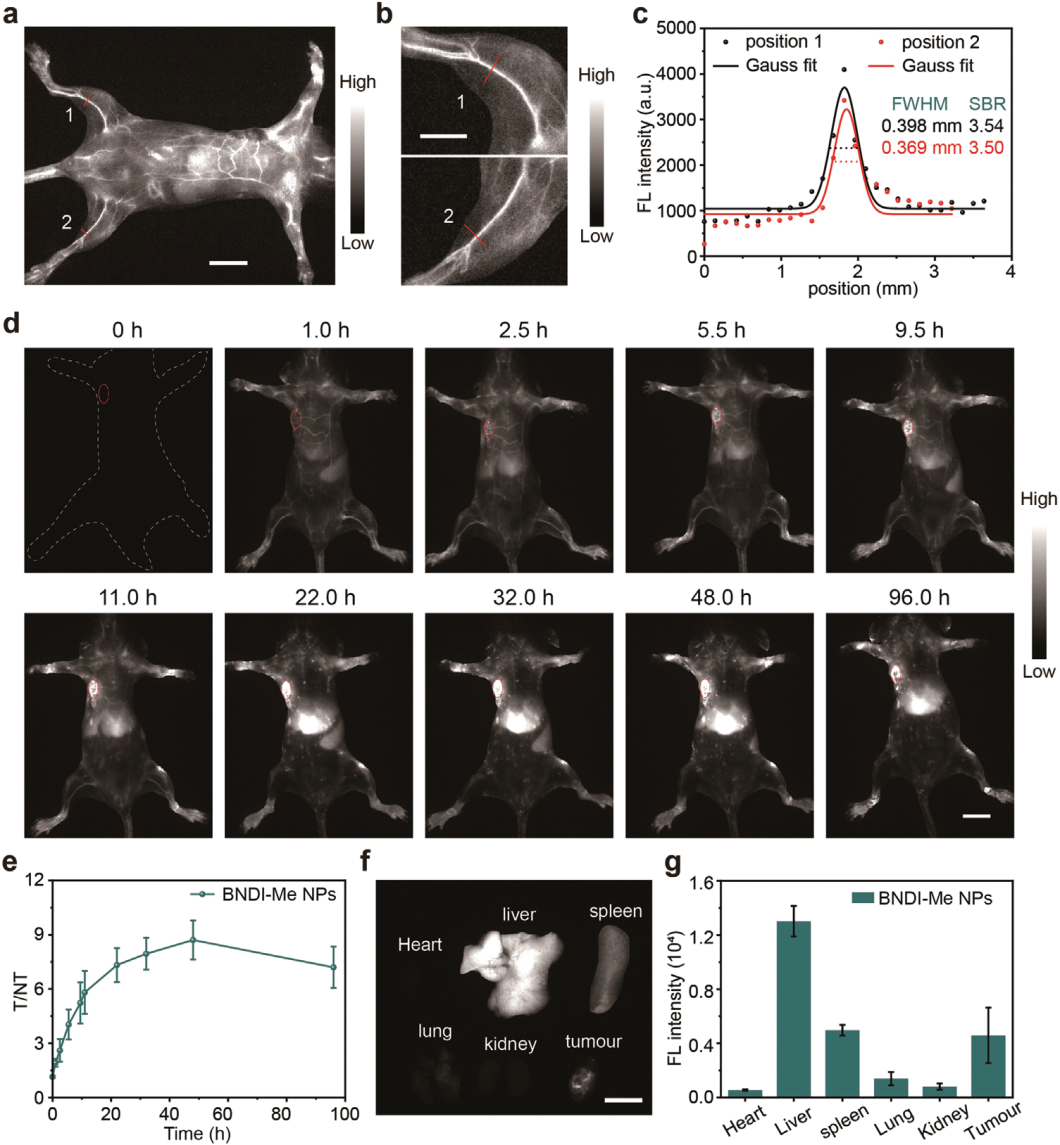
NIR‐II fluorescence imaging of BNDI‐Me NPs in vivo. a) NIR‐II fluorescence images of whole body treated with BNDI‐Me NPs at 5 min post‐injection; scale bar is 10 mm. b) Enlarged picture of hindlimb in (a); scale bar is 5 mm. c) Cross‐sectional intensity profiles (dots) and Gaussian fit (lines) along the red line (hindlimb vasculature) of positions 1, 2 in (b). d) NIR‐II fluorescence images of xenograft 4T1 tumor mice (*n* = 3) at different time points after tail vein injection of BNDI‐Me NPs; scale bar is 10 mm. e) T/NT change based on NIR‐II fluorescence signals over time. Data are plotted as the mean ± SD; *n* = 3. f) NIR‐II fluorescence images of major organs and tumor; scale bar is 10 mm. g) Quantified fluorescence intensity of major organs and tumor. Data are plotted as the mean ± SD; *n* = 3. All experiments were performed under 808 nm laser irradiation using a 1300 nm long‐pass filter to record the fluorescence.

In vivo NIR‐II fluorescence imaging holds great promise for providing deeper tissue penetration, higher spatial resolution compared with the well‐researched NIR‐I fluorescence imaging due to diminished photon scattering and tissue autofluorescence.^[^ ^1a^ ^]^ However, the current development bottleneck in this field is the lack of fluorophores with high brightness.^[^ ^2d^ ^]^ BNDI‐Me NPs show an extremely high NIR‐II fluorescence brightness, which holds great potential for improving fluorescence imaging quality at low systemic injection doses. To evaluate the NIR‐II fluorescence imaging performance of BNDI‐Me NPs in vivo, we first investigate the potential toxicity of BNDI‐Me NPs. PBS and BNDI‐Me NPs (100 µL, 0.5 mg mL^−1^) were intravenously injected into healthy mice. The mice were then sacrificed, and the main organs were extracted at 1, 21 days post‐injection for hematoxylin and eosin (H&E) staining (Figure S21, Supporting Information). Compared with PBS‐treated mice, no obvious differences were observed in the two groups in the main organs, which indicates that BNDI‐Me NPs did not cause significant histological abnormalities or lesions for the main organs. Thus, BNDI‐Me NPs show good biocompatibility and are suitable for in vivo imaging. Next, a considerably low injection dose (100 µL, 0.5 mg mL^−1^) of BNDI‐Me NPs was intravenously injected into 4T1 tumor‐bearing mice to perform NIR‐II fluorescence imaging of tumor in vivo under 808 nm laser excitation. A 1300 nm long‐pass filter was employed to perform the NIR‐II fluorescence imaging because less light absorption and scattering are observed in this window in comparison to the 1000–1300 nm window.^[^ ^1b^ ^]^ Indeed, BNDI‐Me NPs exhibited high fluorescence brightness with 1300 nm long‐pass filter under 808 nm laser irradiation (Figure S22, Supporting Information). Before tail vein injection, almost no NIR‐II fluorescence signals in whole body were observed. Subsequently, NIR‐II fluorescence imaging was performed for the entire body 5 min post‐injection of BNDI‐Me NPs. BNDI‐Me NPs demonstrated bright NIR‐II fluorescence signals in whole body (**Figure** **4**a). Meanwhile, blood vessels exhibited a high signal‐to‐background ratio (SBR = 3.54 and 3.50) and short full width at half maximum (FWHM = 0.398 and 0.369 mm) for the hindlimb vasculature, demonstrating the high spatial resolution imaging feature (Figure 4b,c). Moreover, the hindlimb vasculature was still clearly visualized, even 11 h after the injection of BNDI‐Me NPs, thus realizing long‐term observation for dynamic vasculature (Figure S23, Supporting Information). Additionally, the NIR‐II fluorescence signals in the tumor region gradually increased with time due to the passive targeting of BNDI‐Me NPs via the enhanced permeability and retention effect, and the maximum accumulation was observed with a high tumor‐to‐normal tissue (T/NT) ratio of ≈8.7 in the tumor at ≈48 h post‐injection of BNDI‐Me NPs (Figure 4d,e). In addition, the shape of liver and spleen could be clearly observed (Figure 4d) even in vivo because high fluorescence brightness increases the imaging depth of tissues and resolution ratio, indicating the high‐quality imaging ability of BNDI‐Me NPs. Subsequently, 4T1 tumor‐bearing mice were sacrificed; strong NIR‐II fluorescence signals were observed in the liver and spleen, and relatively poor NIR‐II fluorescence signals were observed in the lung, heart, and kidney, indicating that the hepatobiliary system is the clearance path of BNDI‐Me NPs (Figure 4f,g). These results demonstrate the potential of BNDI‐Me NPs as NIR‐II fluorescent contrast agents for tumor diagnosis even at low systemic injection doses.

### 2.6 Photothermal Properties of Tumor

**Figure 5:**
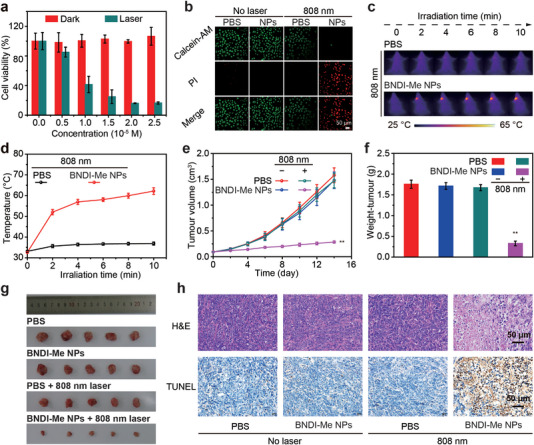
In vitro and vivo PTT effects of BNDI‐Me NPs. a) Relative photo (or dark) viabilities of Hela cells after treatment with BNDI‐Me NPs at different concentrations with (or without) 808 nm laser irradiation (0.3 W cm^−2^) for 10 min. b) Confocal fluorescence images of calcein‐AM (green, live cells) and PI (red, dead cells) co‐stained Hela cells. Control group (only treated with PBS, BNDI‐Me NPs, or 0.3 W cm^−2^ 808 nm laser irradiation), experimental group (BNDI‐Me NPs + 0.3 W cm^−2^ 808 nm laser); scale bar is 50 µm. c) Photothermal images of 4T1 tumor‐bearing mice in different groups within 10 min. d) Temperature changes in the tumor region during laser irradiation. e) Tumor volume growth curves in different groups. f) Average weights and g) images of tumors collected from mice in different groups at the end of PTT. h) H&E staining and TUNEL counterstaining of tumor tissues from mice sacrificed at 14 days post‐treatment; scale bar is 50 µm for H&E and TUNEL staining. The bars are the mean ± s.d., (*n* = 5). **p < 0.01.

PTT is the use of spatiotemporally controllable heat generated from absorbed and transformed non‐invasive light energy of PTT probes to kill tumor cells. Excellent photothermal performance is crucial to irradiation time and laser power. Fortunately, BNDI‐Me NPs incorporate robust NIR‐II fluorescence brightness and photothermal performance, thus BNDI‐Me NPs hold great potential for imaging‐guided PTT to enhance imaging precision and PTT effect with reduced side‐effect at low systemic probe injection doses, weak laser power, and short irradiation time.^[^ ^1^, ^13^ ^]^ To evaluate the photothermal performance of BNDI‐Me NPs in vivo, their phototoxicity and dark toxicity were first analyzed at the cellular level. A 3‐(4,5‐dimethyl‐2‐thiazolyl)‐2,5‐diphenyl‐2‐H‐tetrazolium bromide (MTT) assay was applied to investigate the potential cytotoxicity and PTT effect of BNDI‐Me NPs in Hela and 4T1 cancer cells (**Figure** **5**a, Figure S24, Supporting Information). High cell viability was observed without laser irradiation, indicating their excellent biocompatibility. In contrast, with increased the concentration of BNDI‐Me NPs, the viability of BNDI‐Me NP‐treated Hela and 4T1 cells were reduced under 808 nm laser irradiation (0.3 W cm^−2^) for 10 min. Their viability dropped to ≈16.1% and 13.9% at a concentration of 2.0 × 10^−5^ M, respectively. It demonstrated the high PTT effect of BNDI‐Me NPs. Additionally, live and dead cell staining tests were performed to distinguish between live (green fluorescence) and dead (red fluorescence) cells by co‐staining using calcein‐acetoxymethyl (calcein‐AM) and propidium iodide (PI). As shown in Figure 5b, almost all cells in the control group were stained with green fluorescence. However, in the experimental group with treatment of 808 nm laser, most cells were stained with red fluorescence, which indicates that these were dead cells; this demonstrated the high PTT effect of BNDI‐Me NPs.

The PTT effect of BNDI‐Me NPs in tumor tissues was further evaluated. Mice (*n* = 5) inoculated with xenograft 4T1 tumors were divided into the following four groups: control group (only treated with PBS, BNDI‐Me NPs (100 µL, 0.5 mg mL^−1^), or PBS + 808 nm laser irradiation (0.3 W cm^−2^)) and experimental group (BNDI‐Me NPs (100 µL, 0.5 mg mL^−1^) + 808 nm laser irradiation (0.3 W cm^−2^)). BNDI‐Me NPs (100 µL, 0.5 mg mL^−1^) were injected into 4T1 tumor‐bearing mice through the tail vein. According to the maximum accumulation time of BNDI‐Me NPs obtained during NIR‐II imaging in the tumor, we performed PTT with 808 nm (0.3 W cm^−2^) laser to irradiate the tumor sites at 48 h post‐injection of BNDI‐Me NPs. The temperature variations and thermal imaging of the tumor area during the irradiation process were recorded (Figure 5c). The BNDI‐Me NPs + 808 nm group exhibited rapid temperature growth, rising to ≈62.2 °C within 10 min, which could kill cancer cells forcefully (Figure 5d). In contrast, the PBS + 808 nm group only exhibited slight temperature increase under laser irradiation with a maximum temperature of ≈36.9 °C, which further proved the effective PTT effect of BNDI‐Me NPs. During the next 14 days of PTT, the volume of tumors and body weight of mice in all groups were recorded every 2 days. We observed that the tumor volume in the experimental group decreased significantly as the time passed, with a steady and rapid growth in the control group. This indicates that BNDI‐Me NP‐induced PTT could effectively inhibit tumors (Figure 5e). After 14 days of PTT treatment, the main organs and tumor tissues were removed. The tumor inhibition rate (81.2%) of the BNDI‐Me NPs + 808 nm group was significantly higher than that of other groups (Figure 5f,g), thus demonstrating the high anti‐tumor effect for BNDI‐Me NPs. Immediately thereafter, the cell state and apoptosis of tumor in all groups were studied through hematoxylin‐eosin (H&E) staining and terminal transferase UTP nick‐end labeling (TUNEL) assay; the tumor cells in the experimental group displayed a higher death and apoptosis rate than those in the control group (Figure 5h). Moreover, a steady and slight increase in the body weight of mice was observed in all groups during the entire PTT period, and undamaged main organs were detected by H&E staining, which demonstrated that the PTT was biologically safe (Figures S25 and S26, Supporting Information). In conclusion, BNDI‐Me NPs are an excellent candidate for PTT probes and suitable for PTT of tumor tissues in vivo at low systemic probe injection doses, weak laser power, and short irradiation time.

## 3 Discussion

In this study, we developed a new and highly versatile design strategy to incorporate robust NIR‐II fluorescent brightness and photothermal performance in a single small molecule (BNDI‐Me) by fabricating a large *π*‐conjugated molecule with rigid skeleton and flexible side groups for NIR‐II fluorescence imaging‐guided PTT at a low systemic injection dose. The current popular strategy for the incorporation of high NIR‐II fluorescence brightness and strong photothermal performance involves the combination of various components into one nanoplatform to utilize their respective functions. However, this strategy is not feasible for clinical application owing to its complicated composition and reduced reproducibility.^[^ ^2a^ ^]^ Small organic molecules are preferable for clinical translation owing to their potential biodegradability, simple preparation, and well‐defined molecular structure, which lead to good repeatability. Thus, our strategy to incorporate robust NIR‐II fluorescence brightness and strong photothermal performance in a single molecule is superior to the current popular strategy involving the combination of various components.

In addition, the photophysical mechanisms of *ε* (molecular structure of the ground state), QY (radiative decay of the excited state), and PCE (nonradiative decay of the excited state) are often competitive and interrelated in a single molecule. Therefore, current imaging‐guided PPT probes based on flexible ICT molecular skeleton strategy are often difficult to incorporate robust NIR‐II fluorescence brightness and strong photothermal performance in a single molecule. To overcome this challenge, we provided a solution for this limitation by introducing flexible side groups as steric‐hindrance groups and motion units into a large *π*‐conjugated rigid molecular skeleton to fabricate large *π*‐conjugated molecules with flexibility and rigidity. A large *π*‐conjugated rigid molecular skeleton ensured a high *ε*, which was generally higher than that of commonly used D‐A‐D fluorophores (*ε* = ≈10^3^–10^4^ m ^−1^ cm^−1^) and cyanine dyes (*ε* = ≈10^4^ m ^−1^ cm^−1^) in water.^[^ ^7^ ^]^ Some of these large *π*‐conjugated rigid molecular skeletons with NIR‐I emission demonstrated strong absorption, up to ≈10^8^.^[^ ^8^, ^9^ ^]^ Meanwhile, the flexible side groups could optimize the QY and PCE to simultaneously achieve appreciable values. Therefore, such molecules can incorporate high NIR‐II fluorescence brightness (*ε* × QY) and strong photothermal performance (*ε* × PCE). Compared with recent efforts to improve any one among fluorescence brightness and photothermal performance almost at the expense of other, our strategy boosted light absorption while optimizing the QY and PCE, which assisted the incorporation of high NIR‐II fluorescence brightness and strong photothermal effect in a single molecule, both of which were evidently better than those reported in previous studies. To the best of our knowledge, this is the first study that presents a simple and general strategy for incorporating high NIR‐II fluorescence brightness and photothermal performance in a single molecule. Apart from fluorescent imaging‐guided PTT for cancer, the proposed BNDI‐Me NPs can be further used in imaging‐guided heat‐related bio‐applications, such as target‐specific drug/gene/protein/immune stimulant delivery, intervention of pathological processes, and regulation of biological events.

## Conflict of Interest

The authors declare no conflict of interest.

## Supporting information

Supporting Information

## Data Availability

The data that support the findings of this study are available from the corresponding author upon reasonable request.
