# Supplementary material for: Incorporation of Robust NIR‐II Fluorescence Brightness and Photothermal Performance in a Single Large π‐Conjugated Molecule for Phototheranostics
Source: Adv Sci (Weinh). 2022 Dec 1;10(3):2204695. doi: 10.1002/advs.202204695 (PMC9875648; doi:10.1002/advs.202204695)
Supplement: Supplementary file 1 — Supporting Information [file ADVS-10-2204695-s001.pdf]

ADVANCED SCIENCE
Open Access

Supporting Information

for *Adv. Sci.*, DOI 10.1002/advs.202204695

Incorporation of Robust NIR-II Fluorescence Brightness and Photothermal Performance in a Single Large π-Conjugated Molecule for Phototheranostics

Yuanyuan Li, Yufu Tang, Wenbo Hu, Zhen Wang, Xi Li, Xiaomei Lu, Shufen Chen, Wei Huang and Quli Fan**

Supporting Information

Incorporation of Robust NIR-II Fluorescence Brightness and Photothermal Performance in a Single Large π-Conjugated Molecule for Phototheranostics

*Yuanyuan Li, Yufu Tang**, *Wenbo Hu, Zhen Wang, Xi Li, Xiaomei Lu, Shufen Chen, Wei Huang, Quli Fan**

1. Materials: Unless otherwise noted, all reagents were purchased from J&K Scientific Ltd and Sigma-Aldrich and used without additional purification. All reactions were carried out under nitrogen atmosphere. All other solvents were purchased from Fisher Scientific or Aldrich.

2. Characterization: NMR spectra were recorded on a Bruker Ultra Shield Plus 400 MHz spectrometer (^{1}HNMR 400 MHz and ^{13}C NMR 100 MHz) and referenced to tetramethylsilane (TMS) as the internal standard. Matrix-assisted laser desorption/ionization time of flight mass spectrometry (MALDI-TOF MS, Bruker AutoFlex III system) were carried out to measure mass spectra. The sonicator used for the encapsulation of nanoparticles is a microtip-equipped probe sonicator (VCX 130, SONICS & MATERIALS, INC). Transmission electron microscopy (TEM) imaging was conducted by a HT7700 transmission electron microscope operating at an acceleration voltage of 100 kV. Dynamic light scattering was performed on a particle size analyzer (NanoBrook 90Plus, Brookhaven Instruments Corporation). Absorption data was measured by Shimadzu UV-3600 ultraviolet-visible-near-infrared (UV-Vis-NIR) spectrophotometer. The NIR-II fluorescence spectra were monitored on a commercial NIR-II spectrophotometer (Fluorolog 3, Horiba) equipped with an 808 nm diode laser and an InGaAs NIR detector. The fs-TA spectra were performed with a Newport transient absorption spectrometer, in which a Spectra-physics Tsunami oscillator (800 nm) was used as the seed for a Spectra-Physics Spitre regenerative amplier (1 kHz, 7 mJ). The 3-(4,5-dimethylthiazol-2-yl)-2,5-diphenyltetrazolium bromide (MTT) assay was performed by a PowerWave XS/XS2 microplate spectrophotometer (BioTek, Winooski, VT, USA). Confocal fluorescence imaging was conducted on ZEISS LSM880 laser scanning confocal microscope. Photothermal images were measured by a NIR thermal imager (FLIR E40), and the power density was measured by a VLP-2000 laser power meter. The in vivo and in vitro NIR-II fluorescence imaging

experiments were operated on a NIR-II imaging system (Wuhan Grand-imaging Technology Co., Ltd) with a 1300 nm LP filter under the 808 nm laser irradiation. The NIR-II fluorescence imaging system was equipped with an InGaAs camera (Princeton Instruments).

3. Synthesis

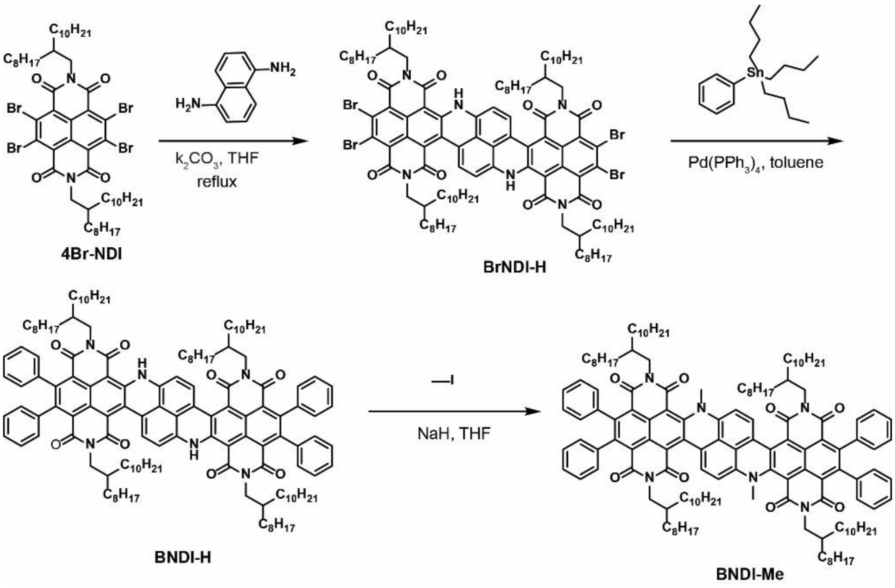

Scheme S1. Synthetic route of BNDI-H and BNDI-Me.

Synthesis of Compound BrNDI-H

4Br-NDI (2.025g, 1.772 mmol), naphthalene-1,5-diamine (0.08 g, 0.506 mmol), K_2CO_3 (0.419 g, 3.036 mmol) were added to a Schlenk tube, and then was vacuumed and purged with dry nitrogen three times. Then, anhydrous tetrahydrofuran (THF) was added, and the mixture were heated to 80°C for 24 h. After the reaction was completed and cooled, the THF was removed by rotary evaporation, and the crude product was extracted with dichloromethane (DCM) and water three times. The organic phase was combined, and dried with anhydrous Na_2SO_4. After removal of the solvent under reduced pressure, the residue was purified by column chromatography on silica gel using PE/DCM (v/v = 4:1 to 1:1) as the eluent to obtain BrNDI-H (0.804g, 75%) as a dark brown solid.

^{1}H NMR (400 MHz, $CDCl_3$, ppm): δ13.86 (s, br, 2H), 7.86 (s, br, 2H), 6.90 (d, *J* = 7.4 Hz, 2H), 4.42 – 3.77 (br, 8H), 2.17-1.80 (br, 4H), 1.49-1.13 (br, 128H), 0.96 – 0.78 (br, 24H).

^{13}C NMR (100 MHz, $CDCl_3$, ppm): δ 164.92, 163.48, 161.29, 159.94, 145.38, 136.80, 132.90, 130.85, 127.84, 125.20, 124.92, 122.35, 121.32, 120.14, 119.80, 113.92, 98.18, 53.42, 46.88, 45.51, 36.55, 36.35, 31.92, 31.80, 31.74, 30.24, 30.22, 30.17, 30.12, 29.75, 29.70, 29.66, 29.62, 29.40, 29.35, 26.61, 22.68, 14.10.

MALDI-TOF MS: 2119.539 (m/z).

Synthesis of Compound BNDI-H

Into a 100 mL two-necked round-bottom flask, BrNDI-H (140 mg, 0.066 mmol), tributyl(phenyl)stannane (1.45 g, 3.96 mmol), and $Pd(PPh_3)_4$ (7.63 mg, 6.6 μmol) were added. The flask was vacuumed and purged with dry nitrogen three times. Then anhydrous toluene (8 mL) was added, and the mixture was heated to reflux and stirred overnight in the dark. After cooling down to room temperature, the reaction mixture was extracted with DCM and water three times. The organic phase was combined, and dried with anhydrous Na_2SO_4. After removal of the solvent under reduced pressure, the residue was purified by column chromatography on silica gel using PE/DCM (v/v = 2:1 to 1:1) as the eluent to obtain BNDI-H (97 mg, 70%) as a dark brown solid.

^{1}H NMR (400 MHz, $CDCl_3$, ppm): δ13.90 (s, 2H), 7.91 (d, J = 8.5 Hz, 2H), 7.21 – 7.11 (m, 12H), 7.06 (d, J = 8.5 Hz, 2H), 6.96-6.78 (br, 8H), 4.01 (dd, J = 12.7, 7.4 Hz, 8H), 1.99-1.82 (br, 4H), 1.33-1.22 (br, 128H), 0.91 – 0.86 (m, 24H).

^{13}C NMR (100 MHz, $CDCl_3$, ppm): δ 166.14, 164.50, 162.88, 161.86, 147.76, 146.10, 144.07, 139.71, 139.35, 137.39, 132.38, 132.18, 129.15, 128.68, 128.44, 127.15, 126.36, 125.89, 123.46, 120.90, 120.17, 113.10, 99.64, 53.44, 45.75, 44.25, 36.39, 36.30, 31.93, 31.58, 31.51, 31.48, 30.16, 30.12, 29.72, 29.68, 29.64, 29.59, 29.37, 26.56, 26.41, 22.70, 14.13.

MALDI-TOF MS: 2108.861 (m/z).

Synthesis of Compound BNDI-Me

BNDI-H (80 mg, 0.038 mmol) and sodium hydride (60% in mineral oil, 30 mg, 0.76 mmol) were added into a 50 mL two-necked flask, then 10 mL dry THF was added, subsequently, the mixture was stirred at room temperature for 30 min under the protection of an argon atmosphere. Then, extreme excess of methyl iodide was added and the resulting mixture was heated to reflux with stirring. Reaction progress was monitored by thin-layer chromatography (TLC) analysis, and methyl iodide was added continuously. After completion, the THF was removed by rotary evaporation and obtained solid residue was extracted three times with DCM and water. The organic phase was dried over anhydrous Na_2SO_4, filtered, and the solvent was then removed by rotary evaporation. The crude product was purified using column chromatography on silica gel using a PE/DCM (v/v = 2:1 to 1:2) as eluent to yield a dark brown solid BNDI-Me (37mg, 45%).

^{1}H NMR (400 MHz, $CDCl_3$, ppm): δ 8.05 (d, J = 8.8 Hz, 2H), 7.27 – 7.06 (m, 14H), 7.01-6.91 (br, 4H), 6.87-6.74 (br, 4H), 4.00 (d, J = 38.0 Hz, 8H), 3.58 (s, 6H), 1.97-1.84 (br, 4H), 1.32-1.20 (br,128H), 0.90-0.86 (br, 24H).

^{13}C NMR (100 MHz, $CDCl_3$, ppm): δ 164.06, 162.82, 161.38, 146.59, 145.56, 144.62, 141.19, 139.79, 133.70, 133.04, 129.25, 129.01, 128.66, 128.51, 127.28, 126.31, 125.66, 123.00, 122.36, 120.46, 117.21, 113.01, 105.66, 45.50, 45.14, 44.60, 36.54, 36.32, 31.93, 31.53, 30.21, 29.73, 29.68, 29.59, 29.37, 26.52, 26.40, 22.69, 14.13.
MALDI-TOF MS: 2136.564 (m/z).

4. DFT Calculations

Density functional theory (DFT) calculations were performed on Gaussian 09 program. The single molecular geometries and the frontier orbital energy levels were obtained by the Becke three-parameter hybrid functional combined with Becke-Lee-Yang-Parr correlation functional (B3LYP) with 6-31G(d) basis sets. Theoretical predictions on all long alkyl substituents were replaced with methyl groups in the calculations.

5. Preparation of nanoparticles (BNDI-H NPs and BNDI-Me NPs)

BNDI-H (1 mg) and F-127 (10 mg/mL) were dissolved in THF (2mL) by sonication. Then, mixed THF solution were used to prepare BNDI-H NPs by rapidly injecting them into distilled-deionized water (10 mL) under continuous sonication with a microtip-equipped probe sonicator (VCX 130, SONICS & MATERIALS, INC) for 2 min, the proportion of the ultrasonic power is set to 50%, and take ultrasound for 8 seconds and stop for 2 seconds as the time interval. After the ultrasound is over, the THF was blowed off with nitrogen under stirring at room temperature. Next, the aqueous solution was filtered through poly (ether sulfone) (PES) syringe driven filter (0.22 μm) (Millipore). Then the resulting solution was concentrated for further experiments. BNDI-Me NPs was prepared by the same method as above.

6. NIR-II fluorescence spectroscopy

The NIR-II fluorescence spectra were measured on a commercial NIR-II spectrophotometer (Fluorolog 3, Horiba). The detection wavelength range of the sample is in the 900 - 1500 nm region when excited by 808 nm laser. The excitation laser beam is pass through the solution sample in a 1 cm path cuvette and the emission was collected with the transmission geometry.

7. Solvatochromic effect

In order to clearly understand the ICT effect, we first measured the absorption and emission spectra of BNDI-H and BNDI-Me in different polar solvents. And then this effect is evaluated quantitatively by the Lippert-Mataga equation:

$$\nu_{abs} - \nu_{em} = \frac{2(\mu_e - \mu_g)^2}{hca^3}\Delta f + count$$

Where μ_g and μ_e are the dipole moment of ground state and excited state, respectively; and ν_{abs} and ν_{em} are the wavenumbers of the maximum absorption and fluorescence, respectively; h is the Planck constant, c is the light velocity, a is the radius of the Onsager cavity, and Δf can be calculated by following equation:

$$\Delta f = \frac{(\varepsilon - 1)}{(2\varepsilon + 1)} - \frac{(n^2 - 1)}{(2n^2 + 1)}$$

where ε is the dielectric constant and n is the refractive index of the solvent. Ploting the Stokes shifts (ν_{abs} - ν_{em}) versus Δf to obtain the slope of the fitted line, and according to the value of *h, c, a*, the difference $\Delta\mu$ between the dipole moments of the excited state and the ground state can be calculated.

8. The measured method of the fluorescence quantum yield (QY)

A stock solution of IR-26 (the reference (QY = 0.5%)) in 1,2-dichloroethane was diluted to obtain a series of samples with absorbance values of ~ 0.10, ~0.08, ~0.06, ~0.04, and ~0.02 at 808 nm measured by UV-Vis-spectroscopy, respectively. And then, the 808 nm laser was used as the exciting light to acquire NIR-II emission spectra of the five solutions in the wavelength region of 900-1500 nm. The integrated fluorescence intensity was plotted against absorbance values at 808 nm and fitted into linear function. The same absorption and emission measurements were performed for BNDI-H NPs and BNDI-Me NPs and in aqueous solutions. According to the calculated slopes of the linear fitting curve, the quantum yields of the samples are calculated with IR-26 as the reference according to the following formula

$$QY_{\text{sample}} = QY_{\text{ref}} \cdot \frac{slope_{\text{sample}}}{slope_{\text{ref}}} \cdot (\frac{n_{sample}}{\text{n}_{\text{ref}}})^2$$

where QY_{ref} is = 0.5 %, n_{sample} and n_{ref} are the refractive index of pure water and 1,2-dichloroethane.

9. Photothermal effect tests of BNDI-H NPs and BNDI-Me NPs

The aqueous solution (0.62 mL) of BNDI-H NPs in eppendorf tubes with different concentrations (0, 2.50× 10^{-6} M, 6.25 × 10^{-6} M, 1.25 × 10^{-5} M, 1.88 × 10^{-5} M, 2.50 × 10^{-5} M) were irradiated by 808 nm laser with power density of 1 W/cm^2 for 10 min. Additionally, the BNDI-H NPs with 2.50 × 10^{-5} M in eppendorf tubes were irradiated by 808 nm laser at different power density (0.25, 0.5, 0.75, 1 W/cm^2) for 10 min. An IR-thermal camera was

utilized to record the temperature change of all sample solutions during irradiation process.

And then photothermal conversion efficiency of BNDI-H NPs was calculated under 808 laser (1 W/cm^2) irradiation. Temperature of BNDI-H NPs solution (2.50 × 10^{-5} M, 0.62 mL) rose to stable level, the laser was turned off and the solution was cooled to room temperature. During the whole process, the temperature of the solution was recorded every 30 seconds. Finally, the photothermal conversion efficiency (PCE) was calculated by following equation:

$$\eta = \frac{(T_{max} - T_{surr})hs - Q_{dis}}{(1 - 10^{-A_\lambda})I}$$

where T_{max} is the maximum stable-state temperature; T_{Surr} is the surrounding temperature; *h* is the heat-transfer coefficient; *s* is the container's surface area; Q_{dis} is the heat dissipation due to the light absorbing of container and water; A_λ is the absorbance value of BNDI-H NPs at 808 nm; and I is the laser intensity (1 W/cm^2). The value of *hs* can be calculated by following equation:

$$hs = \frac{m_D C_D}{\tau_s}$$

where τ_s is the time constant of sample system; and m_D and C_D are the mass (0.62 g) and the heat capacity of water (4.2 J/(g•°C), respectively. The value of τ_s can be calculated by following equation:

$$\tau_s = \frac{t}{-\ln\theta}$$

θ is a dimensionless parameter, known as the driving force temperature. The value of θ can be calculated by following equation:

$$\theta = \frac{T - T_{surr}}{T_{max} - T_{surr}}$$

The BNDI-H NPs (2.50 × 10^{-5} M) solution was performed four laser (808 nm, 1 W/cm^2) on/off cycles to evaluate the photothermal stability, and IR-thermal camera was used to record the temperature curve during the thermal cycle.

The photothermal performance of BNDI-Me NPs was also measured by same method as above.

10. Cell culture

Hela and 4T1 tumour cells were incubated in Dulbecco's Modified Eagle Medium medium

containing 10% fetal bovine serum (FBS) and 1% penicillin/streptomycin at 37 °C in humidified atmosphere of 5% CO_2, and then the cells were collected for the following cell experiments.

11. Animal model

The 4T1-tumour-bearing mice were purchased from Jiangsu KeyGEN Biotech Corp., Ltd. and used in strict accordance with the guideline of the Laboratory Animal Center of Jiangsu KeyGEN Biotech Corp., Ltd., 4T1 cells (2 million) suspended in 50 mL of 50% v/v mixture of Matrigel in supplemented Dulbecco’s modified Eagle’s medium (10% fetal bovine serum, 1% pen/strep (100 IU/mL penicillin and 100 μg/mL streptomycin)) were injected subcutaneously in the right armpit of the mice to establish tumour models in 6-week-old female. Tumours were grown until a single aspect was ~5 mm before being used for in vivo NIR-II fluorescence imaging experiment.

12. NIR-II Fluorescence Imaging of 4T1-Tumour-Bearing Mice

BNDI-Me NPs (100 μL, 0.5 mg/mL) were intravenously injected. NIR-II fluorescence imaging was explored at whole-body vasculature and tumours of living mice upon exposed to 808 nm laser (0.3 W/cm^2) irradiation, and signals were collected by a 1300 nm LP filter. And then, the mice were sacrificed, we further recorded the distribution of BNDI-Me NPs in the main organs, including tumour, heart, liver, spleen, lung and kidney.

13. In vitro photothermal therapy (PTT) efficacy

The MTT assay was used to determine in vitro cytotoxicity of BNDI-Me NPs in HeLa and 4T1 cells with and without laser irradiation. HeLa cells were divided and plated into two 96-well plates. Firstly, the two 96-well plates were randomly divided into two groups for the cytotoxicity test of BNDI-Me NPs: (1) dark cytotoxicity test; (2) PTT test under 808 nm laser excitation. After the cells were cultured for 24h, the culture medium was removed, different concentrations of BNDI-Me NPs solutions were added to the two 96-well plates and cultured for 12 hours. The concentration gradient of BNDI-Me NPs in each 96-well plate is 0, 5.0×10^{-6} M, 1.0×10^{-5} M, 1.5×10^{-5} M, 2.0×10^{-5} M, 2.5×10^{-5} M. Then the PTT group was irradiated with 808 nm laser. After 5 hours, 10 μL MTT (0.5 mg/mL) solution was added to each well. After 4 h incubation at 37 °C, the supernatant was removed and 200 μL dimethyl sulfoxide (DMSO) was added. A PowerWave XS/XS2 microplate spectrophotometer was used to record the absorbance intensity at 490 nm, and the cellular viability relative to the control group was calculated as $A_{sample}/A_{control}$, in which A_{sample} and $A_{control}$ represent the

average absorption of group treated with BNDI-Me NPs and untreated group, respectively. 4T1 cells were treated with same process.

14. Live-dead cell staining

For calcein AM/ PI tests, the HeLa cells were divided into four groups: only treated with PBS or BNDI-Me NPs, only 808 nm laser irradiation, treated with BNDI-Me NPs + 808 nm laser irradiation. And then PTT groups were irradiated by corresponding laser for 10 min, laser power density is 0.3W/cm^2. Then, the HeLa cells were incubated for 4 h. After staining with the mixture of Calcein AM/PI for 20 min and washing with PBS twice, images of the four samples were taken by ZEISS LSM880 laser scanning confocal microscope.

15. PTT Efficacy of BNDI-Me NPs on 4T1-Tumour-Bearing Mice

BALB/c mice were randomly divided into four groups (n = 5): Control (only treated with PBS, BNDI-Me NPs, or PBS + 808 nm laser irradiation), experimental group (BNDI-Me NPs + 808 nm laser irradiation), laser power density is 0.3 W/cm^2. The concentration of BNDI-Me NPs injected into the mice is 0.5 mg/mL, and the injection dose in every mouse is 100 μL. And then, we performed PTT on the tumour sites of mice in laser groups with 808 nm laser irradiation for 10 min at about 48 h post-injection of BNDI-Me NPs. Then during the next 14 days of PTT, the weights of mice and the volumes of tumours were measured every two days. The tumour volumes were measured and calculated based on the following formulae, e.i., tumour volume = (length × width^2)/2. The mice were sacrifaced after 14 days, and the major organs (heart, liver, spleen, lung, kidney and tumour) were extracted and cell death and apoptosis levels were performed H&E staining and the TUNEL staining assay.

16.Statistical analysis

Data were presented as mean ± SD. The statistical significance of differences among the groups tested was determined using one-way ANOVA. $^{**}p < 0.01$ was considered statistically significant.

17. Supplementary Figures and tables

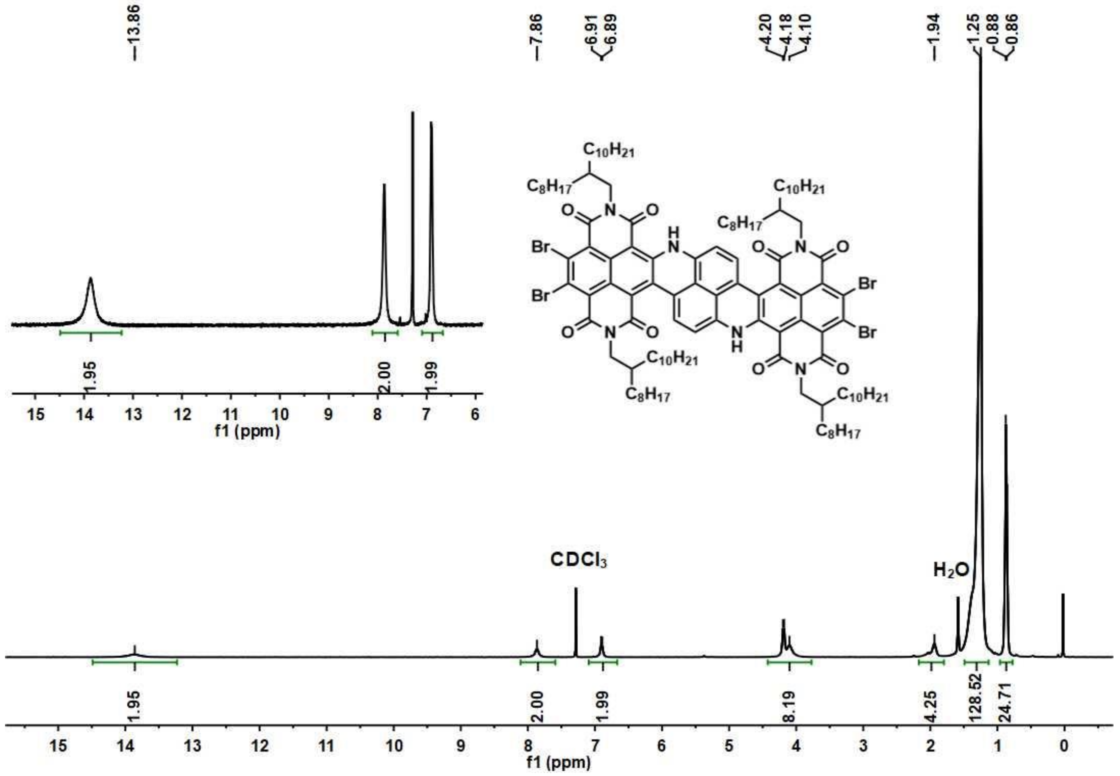

Figure S1. ^{1}H NMR Spectrum of BrNDI-H in $CDCl_3$.

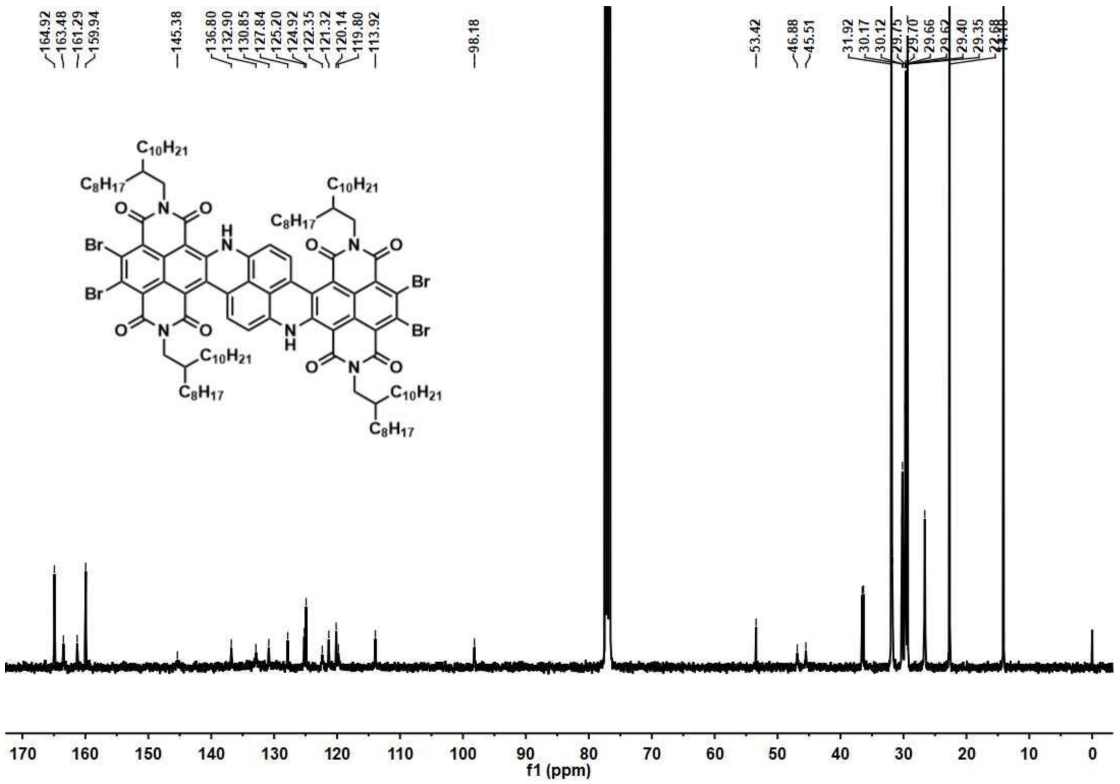

Figure S2. ^{13}C NMR Spectrum of BrNDI-H in $CDCl_3$.

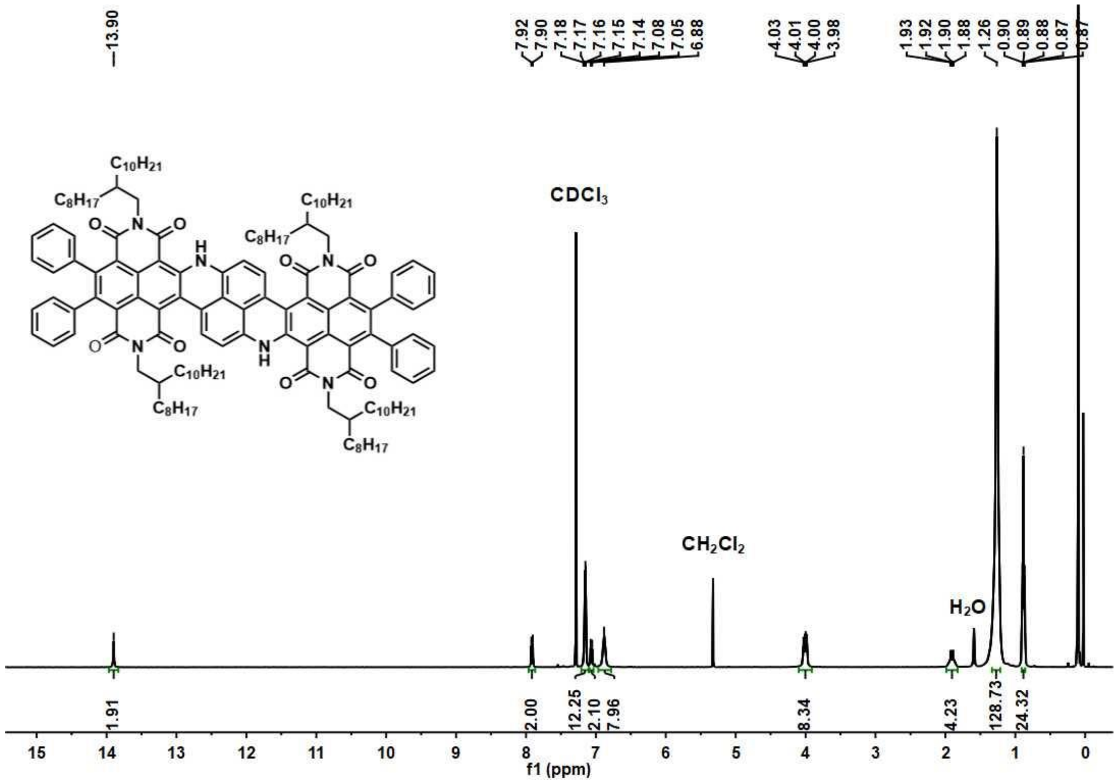

Figure S3. ^{1}H NMR Spectrum of BNDI-H in $CDCl_3$.

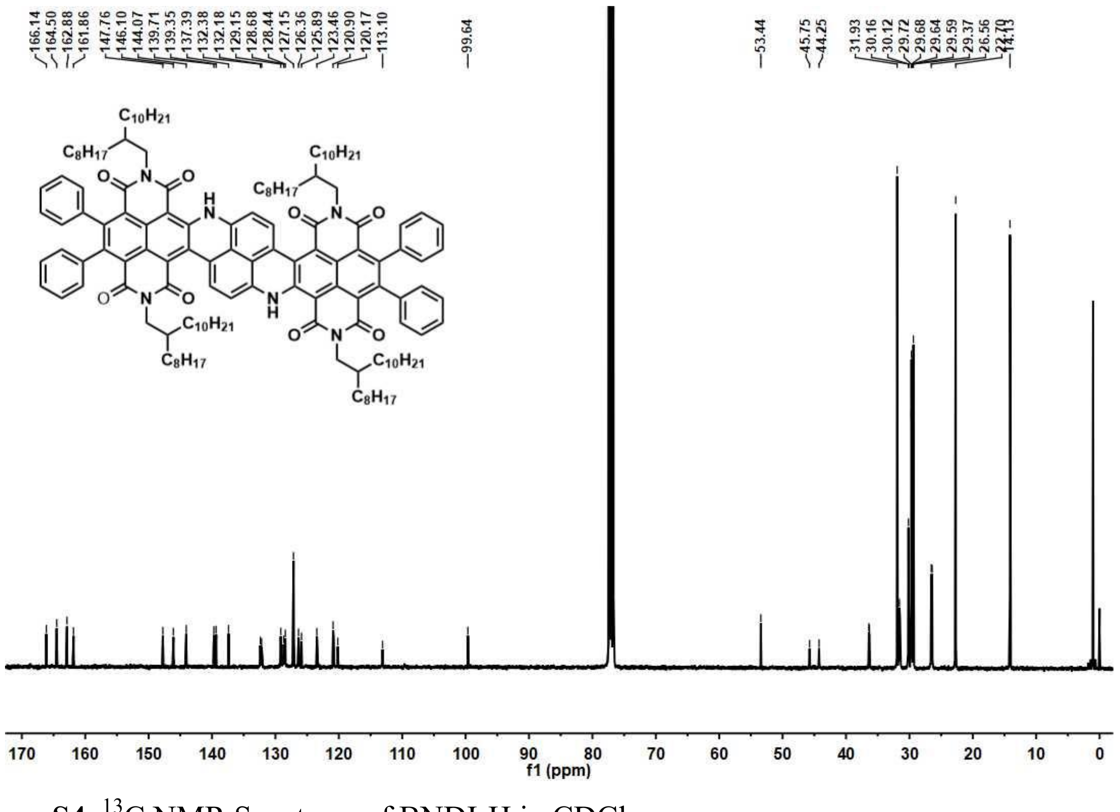

Figure S4. ^{13}C NMR Spectrum of BNDI-H in $CDCl_3$.

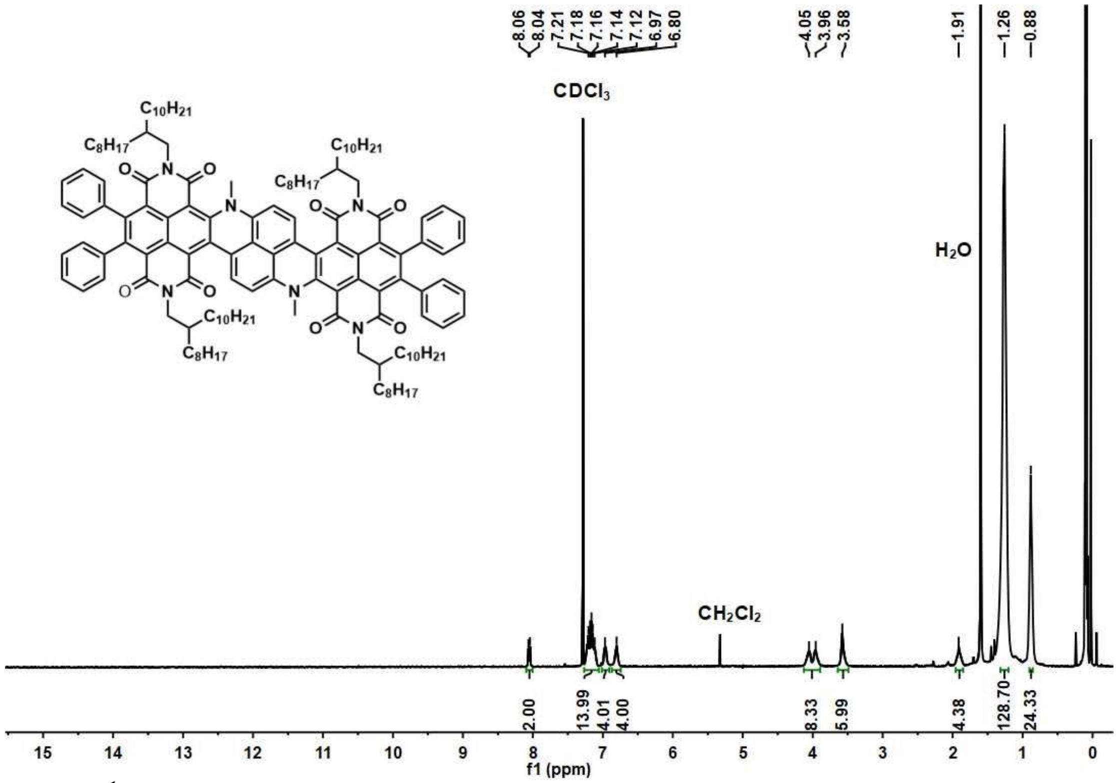

Figure S5. ^{1}H NMR Spectrum of BNDI-Me in $CDCl_3$.

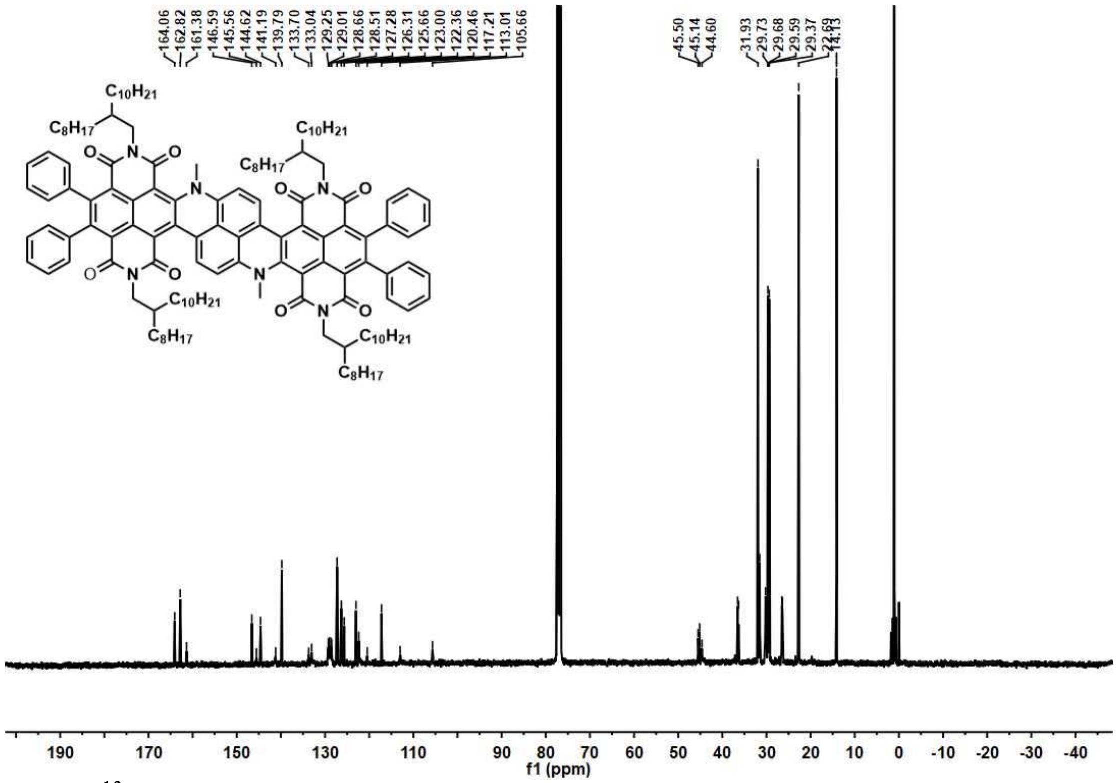

Figure S6. ^{13}C NMR Spectrum of BNDI-Me in $CDCl_3$.

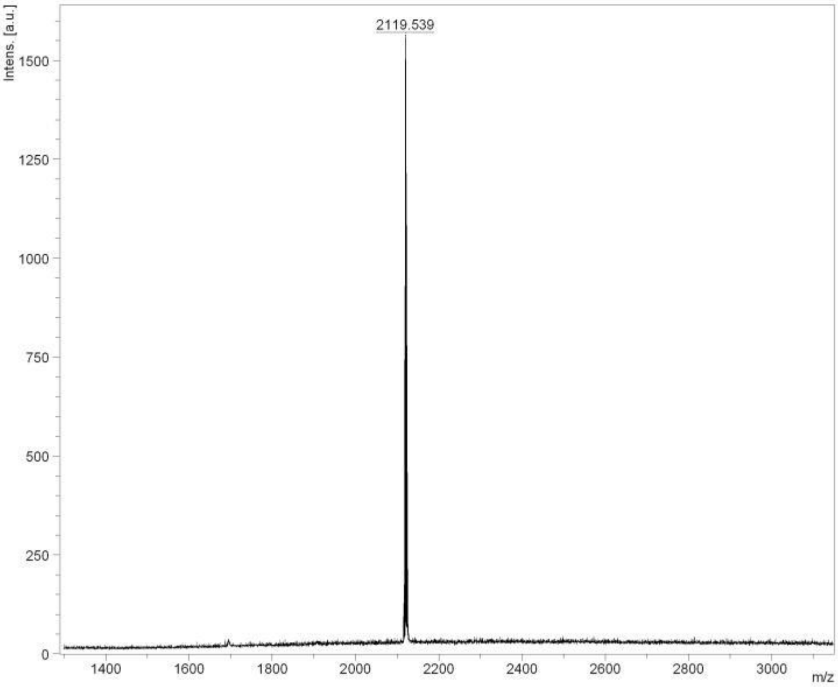

Figure S7. The Matrix assisted laser desorption/ionization time-of-flight (MALDI-TOF) mass spectrum of BrNDI-H.

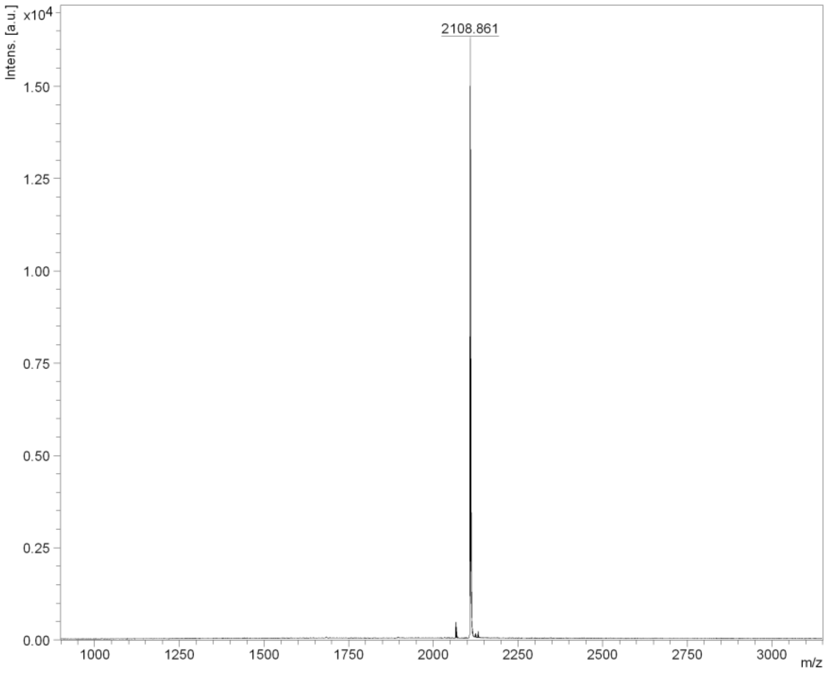

Figure S8. The Matrix assisted laser desorption/ionization time-of-flight (MALDI-TOF) mass spectrum of BNDI-H.

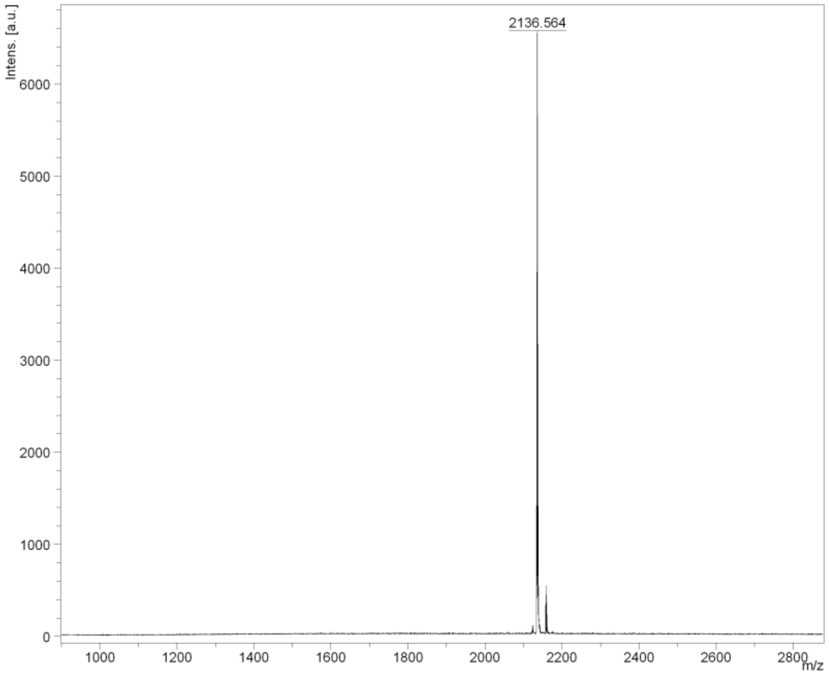

Figure S9. The Matrix assisted laser desorption/ionization time-of-flight (MALDI-TOF) mass spectrum of BNDI-Me.

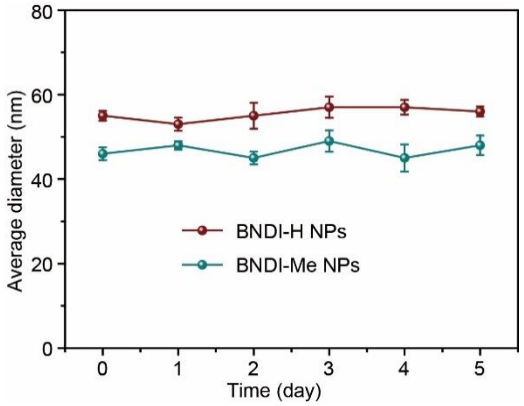

Figure S10. Average hydrodynamic diameters of BNDI-H NPs and BNDI-Me NPs in aqueous solution for different time (mean ± sd, n = 3).

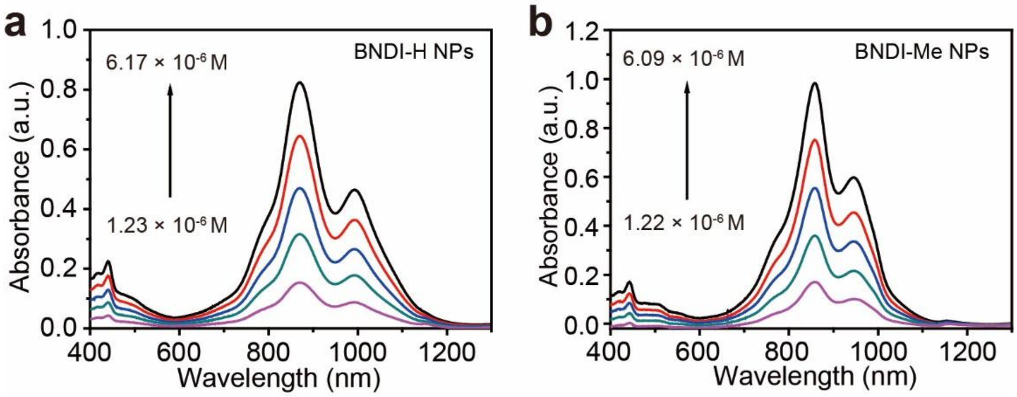

Figure S11. The absorption spectra of (a) BNDI-H NPs and (b) BNDI-Me NPs in pure water

under various concentration.

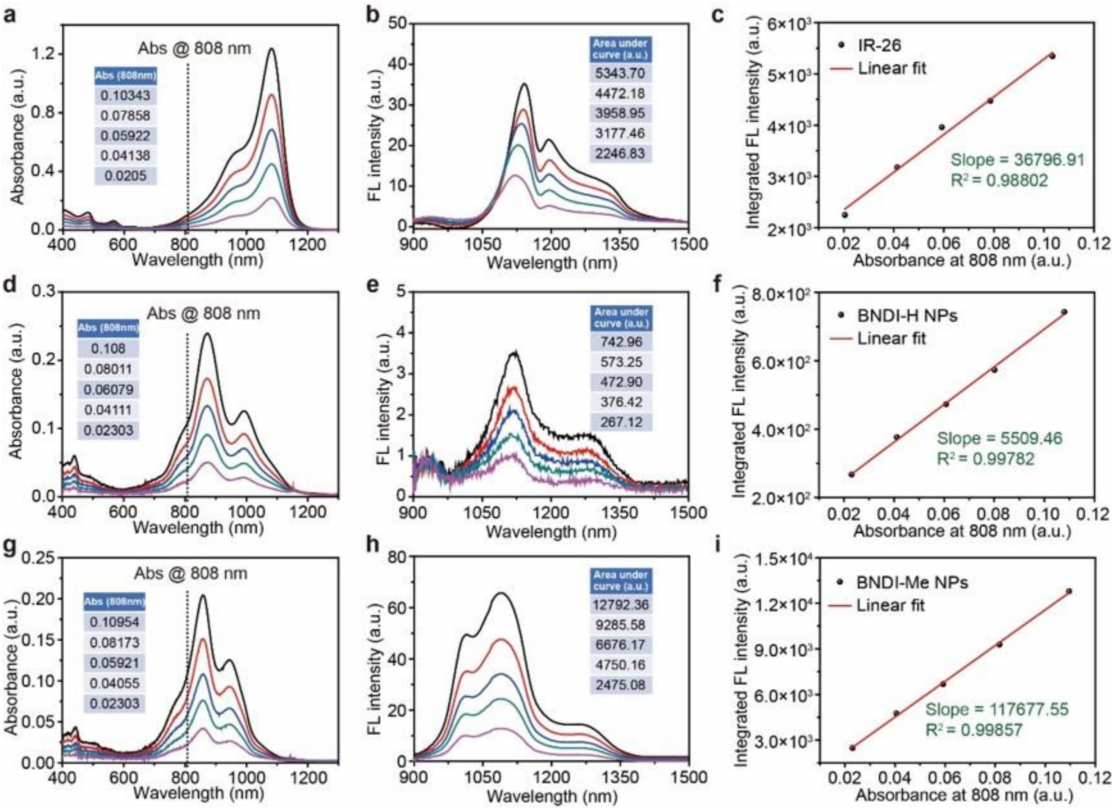

Figure S12. Fluorescence QYs of BNDI-H NPs and BNDI-Me NPs in aqueous solutions. (a) UV-Vis-NIR absorption spectra of IR-26 in 1,2-dichloroethane (DCE) with absorbance values at 808 nm of ~0.10 (Black), ~0.08 (red), ~0.06 (Blue), ~0.04 (dark cyan) and ~0.02 (purple), respectively. The exact absorbance of each solution was listed in the inset table. (b) NIR-II emission spectra of five solutions in (a). Their fluorescence emission spectra in the 900 - 1500 nm range was taken under 808 nm laser excitation, shown in black, red, blue, dark cyan and purple line, respectively. Area under curve in the emission spectrum for each solution was then calculated and listed in the inset table. (c) For all DCE solutions of IR-26, their absorbance values were then plotted versus area under curve in the emission spectrum, and fitted into a linear function, where slope of the fitted line was read as shown. The same absorption and emission measurements, linear fitting curves were performed for BNDI-H NPs (d - f) and BNDI-Me NPs (g - i) in aqueous solutions.

The change in NIR-II fluorescence intensity of BNDI-Me NPs in serum was measured under continuous 808 nm laser irradiation (1 W cm^{-2}) for 30 min. As shown in Figure S13, the fluorescent intensity of BNDI-Me NPs shows negligible attenuation within 30 min, exhibiting excellent photostability in serum.

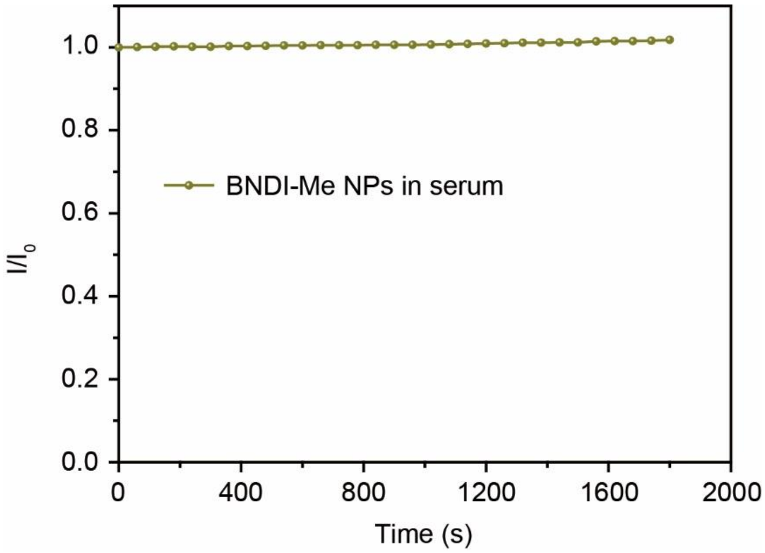

Figure S13. Photostability of BNDI-Me NPs (2.50×10^{-5} M) in serum upon irradiation of continuous 808 nm (1 W cm^{-2}) laser for 30 min. I/I_0 is the ratio of the mean fluorescence intensity of the samples after radiation to that of the original mean fluorescence intensity.

The relative NIR-II fluorescence intensity of both nanoparticles (2.50×10^{-5} M) at different temperatures (25, 30, 35, 37.5, 40, 45 ℃) in aqueous solution compared to fluorescence intensity at 25 ℃ was recorded. Figure S14 shows no obvious fluorescence change with the temperature change observed compared to fluorescence intensity at 25 ℃, demonstrating their high structural stability at different body temperatures.

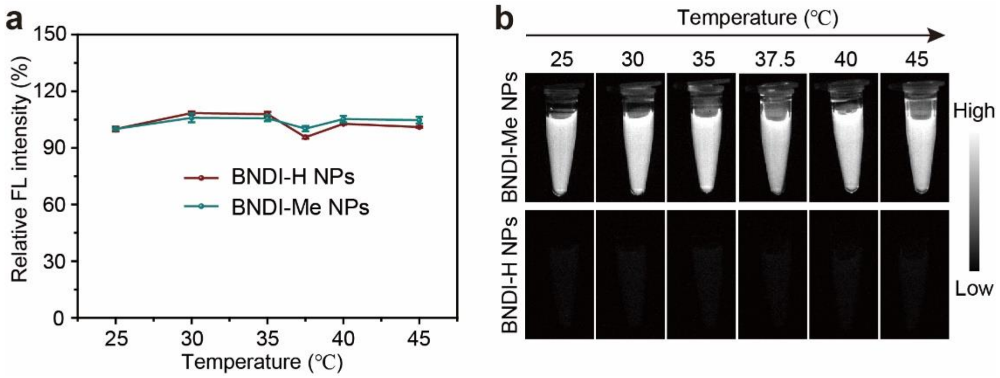

Figure S14. The relative NIR-II fluorescence intensity of BNDI-H and BNDI-Me NPs at different temperatures (25, 30, 35, 37.5, 40, 45 ℃) in aqueous solution compared to fluorescence intensity at 25 ℃. Data represent mean value ± standard deviation (n = 3). (c) The corresponding NIR-II images at different temperatures.

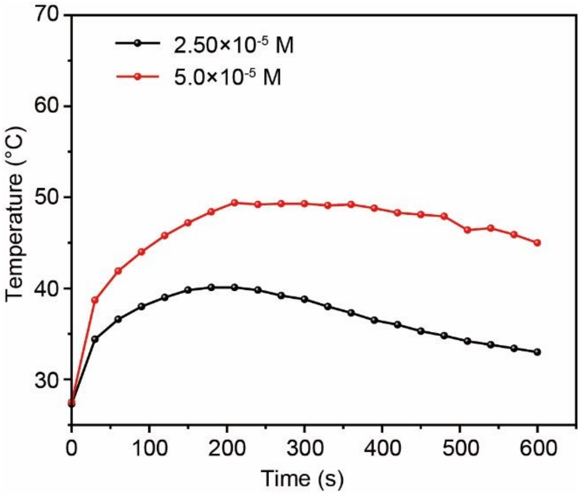

Figure S15. Temperature change curves of indocyanine green (ICG) (2.50×10^{-5} M) and ICG (5.0×10^{-5} M) under 1 W/cm^2 808 nm laser irradiation within10 min.

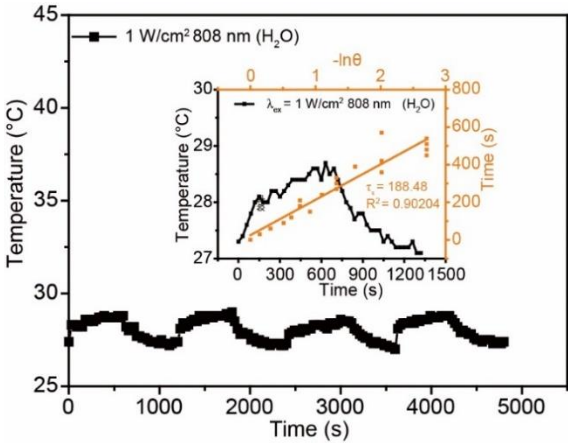

Figure S16. Temperature variation curves of pure water over four cycles under laser on/off of 808 nm with a power density of 1W/cm^2. The insets are the photothermal performances of pure water under 808 nm laser irradiation. The samples were irradiated to reach a temperature plateau and then the laser was shut off. Temperature variations were monitored by thermal imaging camera. Plot of linear time data from cooling time versus the negative natural logarithm of the temperature driving force obtained from the cooling curve. The slope represents the sample system time constant. (τ_s).

Furthermore, we studied the absorption spectra change of BNDI-H NPs (Figure S17a) and BNDI-Me NPs (Figure S17b) through continuous 808 nm (1 W cm^{-2}) laser irradiation for 30 min. The absorption spectra and solution color exhibited negligible change. However, the absorption of clinically popular ICG aqueous solution significantly decreased, and the color of the solution shifted from green to pale yellow (Figure S17c). Above all, excellent photostability impelled BNDI-Me NPs as a promising candidate for PTT in *vivo*.

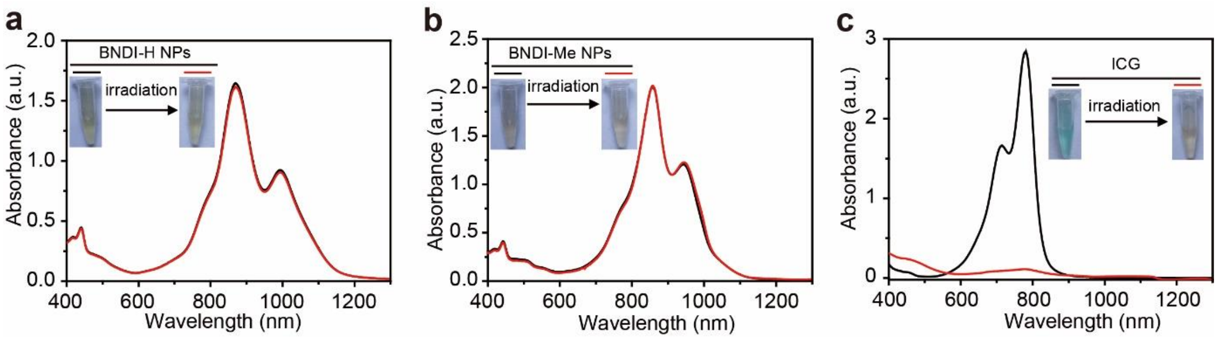

Figure S17. Absorbance stability of (a) BNDI-H NPs, (b) BNDI-Me NPs and (c) ICG solution under irradiation of 808 nm laser (1 W/cm^2, 30 min). The insets are photos of (a) BNDI-H NPs, (b) BNDI-Me NPs and (c) ICG solution before (left) and after (right) laser irradiation.

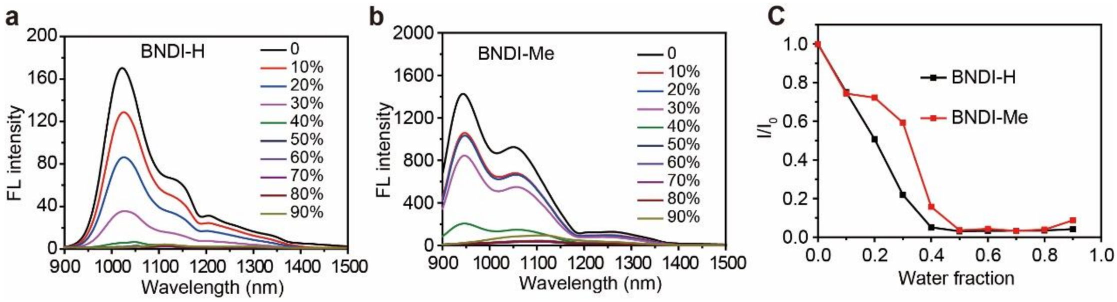

Figure S18. The fluorescence spectrum of (a) BNDI-H and (b) BNDI-Me in THF contained different water fraction. (c) Change in fluorescence intensity with water fraction in water/THF mixtures.

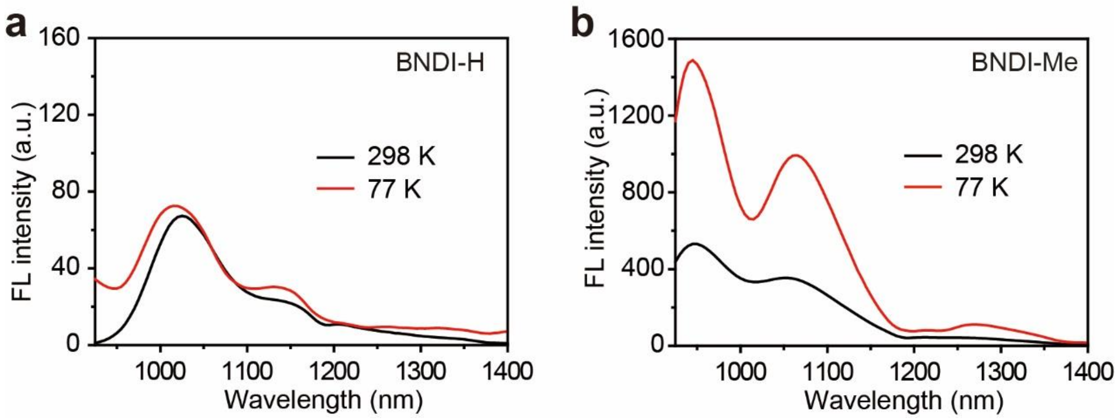

Figure S19. Fluorescence intensity of BNDI-H (a), BNDI-Me (b) in THF solution at 298 K and 77 K.

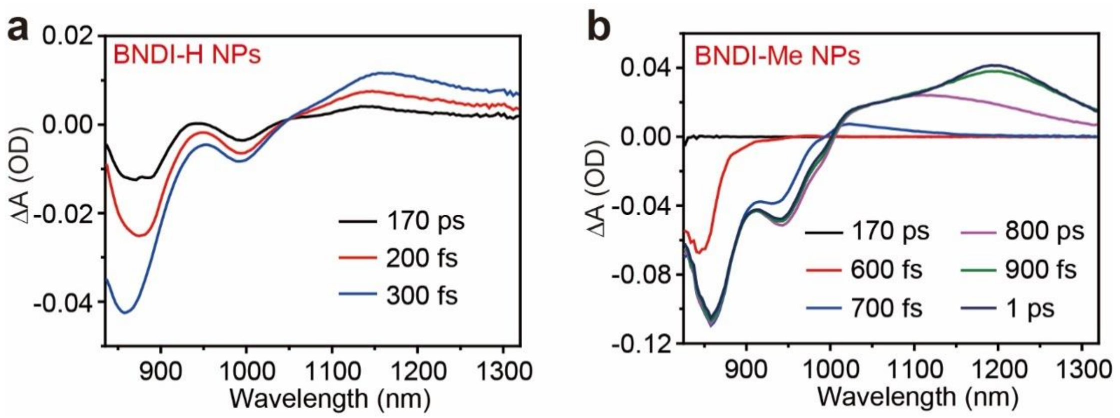

Figure S20. Fs-TA spectra of BNDI-H NPs (a) and BNDI-Me NPs (b) at different delay times.

To investigate the potential toxicity of BNDI-Me NPs, we used healthy mice to investigate haematoxylin and eosin (H&E) staining analysis postinjection. The control was performed the same treatment except using PBS. The histological analyses were performed at time points of 1, 21 days postinjection, respectively. The mice were then sacrificed. The heart, lung, liver, spleen and kidney were embedded in paraffin, sectioned, and stained with hematoxilin and eosin, respectively. Then subsequently processed for histopathological examination under light microscope. No significant damage was detected by H&E staining in main organs during the whole-treatment period of both groups, which demonstrated no apparent histological abnormalities or lesions in the BNDI-Me NP-treated group (Figure S21).

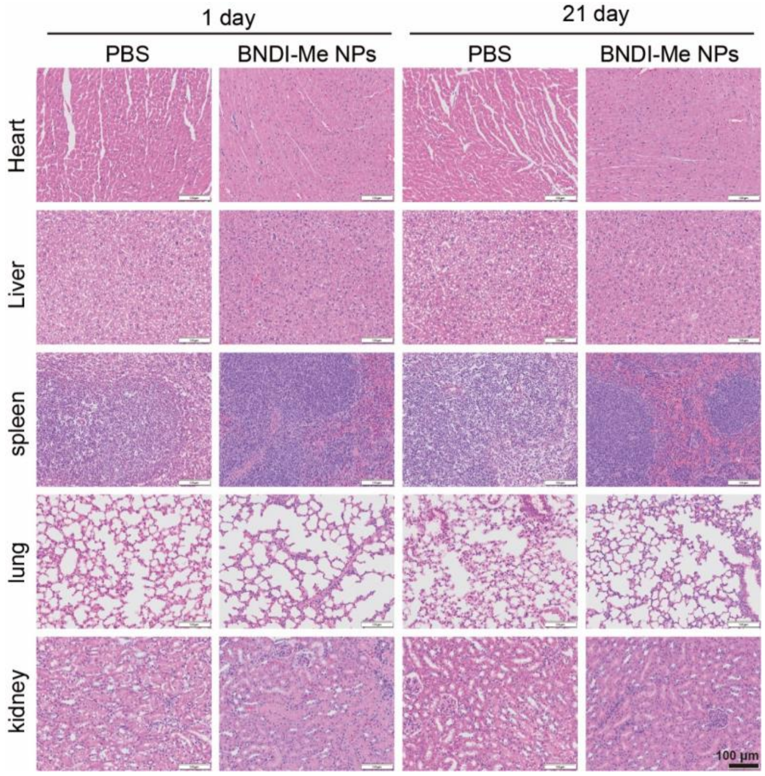

Figure S21. Histological data (haematoxylin and eosin stained images) obtained from the heart, liver, spleen, lung and kidney of the BNDI-Me NPs or PBS-treated mice at 1, 21 days post-injection (scale bars: 100 μm for all panels).

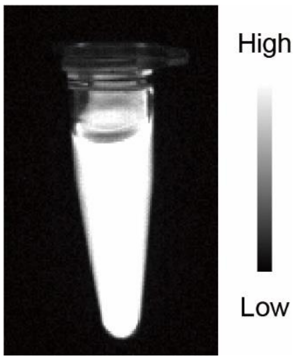

Figure S22. NIR-II fluorescence image of BNDI-Me NPs (5.0×10^{-5} M) under 808 nm laser irradiation with LP1300 nm filter (exposure time: 100 ms).

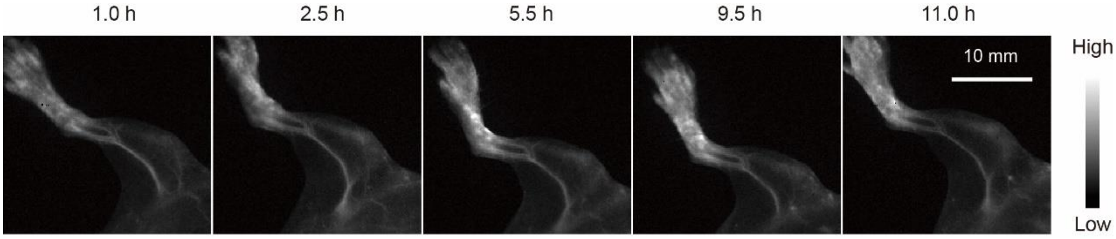

Figure S23. NIR-II fluorescence imaging in hindlimb vasculature of living mouse after injected with BNDI-Me NPs (1300 nm LP filter) over time. Scale bar is 10 mm.

We supplemented the dark and phototoxicity of BNDI-Me NPs in 4T1 cells. As shown in figure S24, high cell viability was observed without laser irradiation, indicating their excellent biocompatibility. In contrast, the viability of BNDI-Me NP-treated 4T1 cells were reduced when the concentration was increased under 808 nm laser irradiation (0.3 W cm^{-2}) for 10 min, which dropped to approximately 13.9% at a concentration of 2.0×10^{-5} M, demonstrating the high photothermal effect of BNDI-Me NPs.

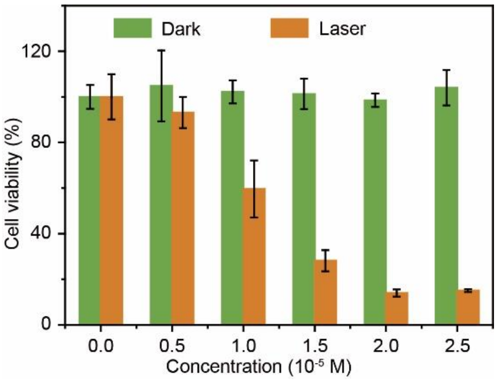

Figure S24. 4T1 cell viability incubation with BNDI-Me NPs of different concentrations with (or without) 808 nm laser irradiation (0.3 W/cm^2) for 10 min (mean ± sd, n = 5).

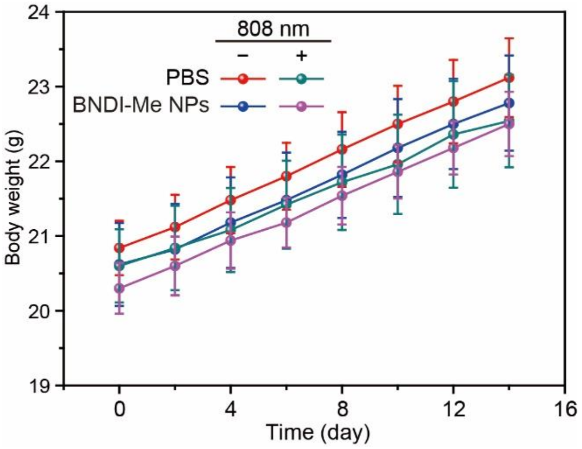

Figure S25. Body weight curves of mice in different groups during the PTT period.

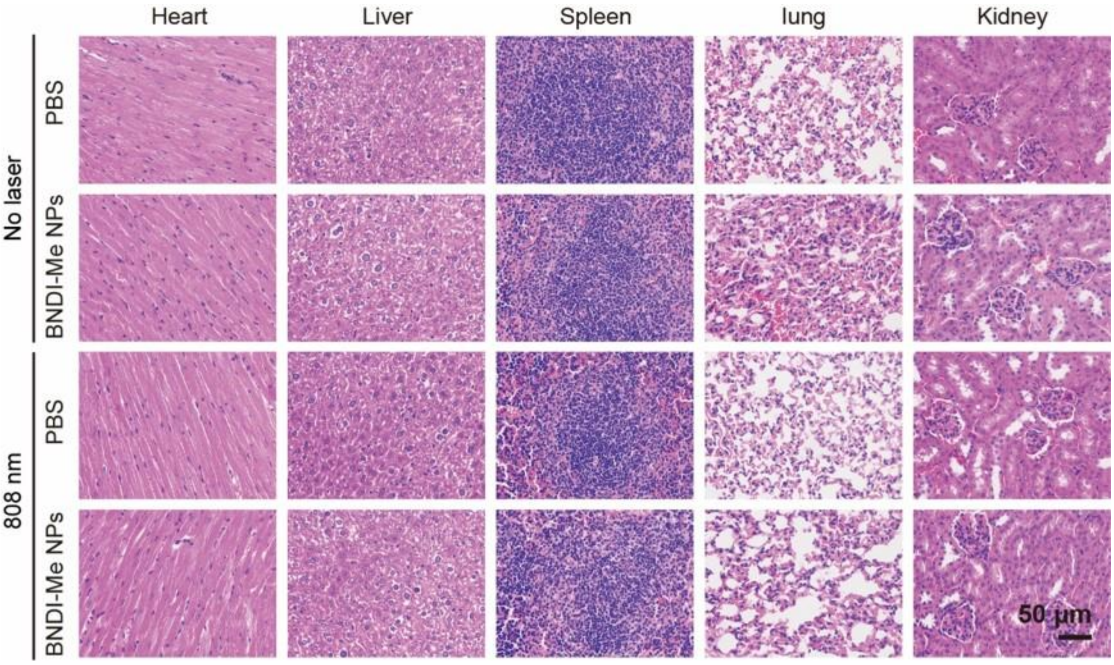

Figure S26. H&E analysis of the major organs collected from different groups after treatment (scale bar 50 μm).

Table S1. Photophysical characterization and photothermal effect of previously reported organic NIR-II fluorophores and high-efficiency organic photothermal agents in aqueous solution.

Number	Name	ε[a] (M^{-1} cm^{-1})	QY[b] (%)	PCE	Brightness (ε × QY)	ε × PCE	Ref.
1	BNDI-Me NPs	1.64×10^5 (859 nm, λ_{max})	1.4	50%	2296	8.20×10^4	This work
		8.83×10^4 (808 nm, laser)			1236.2	4.42×10^4	
2	COTIC-4F NPs	8.70×10^4 (934 nm, λ_{max})	0.35	N/A	304.5	N/A	[1]
		6.80×10^4 (808 nm, laser)			238		
	CBTIC-4F NPs	8.80×10^4 (949 nm, λ_{max})	1.4		1232		
		6.70×10^4 (808 nm, laser)			938		

3	L1013 NPs	1.39×10^4 (761 nm, λ_{max})	9.9	N/A	1376.1	N/A	2
4	L897 NPs	1.0×10^4 (715 nm, λ_{max})	5.8	N/A	580	N/A	3
5	TB1 NPs	1.21×10^4 (740 nm, λ_{max})	6.2	N/A	750.2	N/A	4
6	IR-BEMC6P	1.31×10^3 (725nm, λ_{max})	1.8	N/A	23.58	N/A	5
	IR-BGMC6P	2.42×10^3 (737nm, λ_{max})	1.5		36.3		
7	TTQP NPs	3.29×10^4 (711 nm, λ_{max})	4.7	N/A	1546.3	N/A	6
8	IR-FP8P	1.3×10^4 (748 nm, λ_{max})	6	N/A	780	N/A	7
9	IR-FTAP	5.0×10^3 (733 nm, λ_{max})	5.3	N/A	265	N/A	8
10	IR-FEP	5.69×10^3 (780 nm, λ_{max})	2.0	N/A	113.8	N/A	9
	IR-FTP	6.95×10^3 (828 nm, λ_{max})	0.02		1.39		
11	HL3 dots	9.3×10^3 (750 nm, λ_{max})	11.7	N/A	1088.1	N/A	10
12	TT1-*o*CB NPs	1.54×10^4 (732 nm, λ_{max})	8.6	N/A	1324.4	N/A	11
		1.13×10^4 (793 nm, laser)			971.8		
	TT2-*o*CB NPs	1.93×10^4 (752 nm, laser)	7.8		1505.4		
		1.62×10^4 (793 nm, laser)			1263.6		
	TT3-*o*CB NPs	2.07×10^4 (784 nm, λ_{max})	4.6		952.2		
		2.02×10^4 (793 nm, laser)			929.2		
13	TTQiT NPs	3.89×10^4 (755 nm, λ_{max})	3.7	N/A	1439.3	N/A	12
		3.66×10^4 (808 nm, laser)			1354.2		
	TTQT NPs	3.77×10^4 (730 nm, λ_{max})	2.6		980.2		
		2.57×10^4 (808 nm, laser)			668.2		
	TTQPL NPs	3.72×10^4 (745 nm, λ_{max})	1.5		558		
		3.12×10^4 (808 nm, laser)			468		
14	FD-1080 J-aggregate	5.0×10^4 (1028 nm, λ_{max})	0.54	N/A	270	N/A	13
15	TPA-Et	5.31×10^4 (764 nm, λ_{max})	0.04(micelles) [c]	52.5 %	21.24	2.79×10^4	14
16	LZ-1060	1.08×10^5 (1021 nm, λ_{max})	0.6	N/A	648	N/A	15
	LZ-1092	1.06×10^5 (1035	0.2		212		

		nm, λ_{max})					
	LZ-1105	1.01×10^5 (1040 nm, λ_{max})	0.3		303		
	LZ-1118	1.08×10^5 (1052 nm, λ_{max})	0.1		108		
17	MB	7.12×10^4 (665 nm, λ_{max})	0.2	N/A	142.4	N/A	16
18	ICG	1.2×10^5 (785 nm, λ_{max})	1	N/A	1200	N/A	17
19	CX-3	5.71×10^4 (DMSO, 1089 nm, λ_{max})	0.82	N/A	468.22	N/A	18
20	pNIR-4	5.73×10^3 (THF, 709nm, λ_{max})	2.2	N/A	126.1	N/A	19
21	5H5	3.42×10^4 (MeCN, 1069 nm, λ_{max})	2.6	N/A	889.2	N/A	20
22	BDTR9-OC8 NPs	6.15×10^4 (757 nm, λ_{max})	0.41	77%	252.15	4.74×10^4	21
	BDTR9-C8 NPs	7.03×10^4 (766 nm, λ_{max})	1.89	69%	1328.67	4.85×10^4	
23	BPN-BBTD NPs	~2.54×10^4 (700 nm, λ_{max})	1.8	39.8 %	457.2	1.01×10^4	22
		1.27×10^4 (785 nm, laser)			228.6	5.05×10^3	
24	PorCP NPs	4.23×10^4 (800 nm, λ_{max})	N/A	63.8 %	hardly detecta-ble	2.70×10^4	23
25	SPNV	3.21×10^4 (819nm, λ_{max})	N/A	71%	N/A	2.28×10^4	24
26	WMG1	3×10^4 (820nm, λ_{max})	N/A	60%	No fluores-cence	1.8×10^4	25
	WMG2	4×10^4 (753nm, λ_{max})		54%		2.16×10^4	
27	PBDT-DIID NPs	5.25×10^4 (786 nm, λ_{max})	N/A	70.6 %	no fluorescen t signals	3.71×10^4	26
28	BH 990	1.11×10^5 (MeOH, 967 nm, λ_{max}) [d)]	N/A	50.9 %	Almost no fluorescen ce	5.65×10^4	27
29	BAF3 NPs	~ 5.0×10^3 (907 nm, λ_{max})	<0.1 %[e)]	47%	<5	2.35×10^3	28
	BAF4 NPs	~ 7.5×10^3 (1003 nm, λ_{max})		80%	<7.5	6×10^3	
30	Cy7-TCF nanoaggre gates	2.76×10^4 (730 nm, λ_{max})	N/A	62.3 %	N/A	1.72×10^4	29
31	IR-SS	1.77×10^4 (THF, 1060 nm, λ_{max})	N/A	77%(NPs)	N/A	1.36×10^4	30
32	2TPE-2NDTA	6.78×10^4 (THF, λ_{max})	N/A	54.9(NPs)%	N/A	3.72×10^4	31
33	A1	4.761×10^4 (THF, λ_{max})	1.23(NPs)	55.3(NPs)%	585.6	2.63×10^4	32

34	BPBBT NPs	~ 2.7 × 10^4 (700 nm, λ_{max})	1.45	27.5 %	391.5	7.43 × 10^3	33
		9 × 10^3 (808 nm, laser)			130.5	2.48 × 10^3	
35	MAPE-NPS	~ 6.1× 10^4 (808 nm, laser)	0.178 [c)]	N/A	108.6	N/A	34
		~ 6.8× 10^4 (856 nm, λ_{max})		40.7 %	121.04	2.77 × 10^4	
		3.4 × 10^4 (1064 nm, laser)	N/A		N/A	1.38 × 10^4	
36	TA1 NPs	2.14 × 10^4 (685 nm, λ_{max})	0.08[f)]	84.8 %	17.12	1.81 × 10^4	35
		5.58 × 10^3 (808 nm, laser)			4.46	4.73 × 10^3	
37	NJ-1060 NPs	1.26 × 10^4 (882 nm, λ_{max})	0.1	21.4 %	12.6	2.7 × 10^3	36
	CCNU-1060 NPs	1.60 × 10^4 (877 nm, λ_{max})	0.3	26.5 %	48	4.24 × 10^3	

[a)] The molar extinction coefficients were directly obtained or calculated based on the data provided from absorbance spectra or references. [b)] The QY was re-calculated using QY of IR-26 = 0.5% in DCE as a standard. [c)] Absolute QY. [d)] The ε of cyanine in water is attenuated (~ 10^4 M^{-1} cm^{-1}) than that in organic solution, generally, we choose ε of methanol with high polarity provided in the reference. [e)] IR-26 (Φ_f = 0.1% in DCE) for BAF3-4 as reference. [f)] ICG (QY = 1%) in water were used as the reference for calculating the fluorescence QY of TA1 NPs.

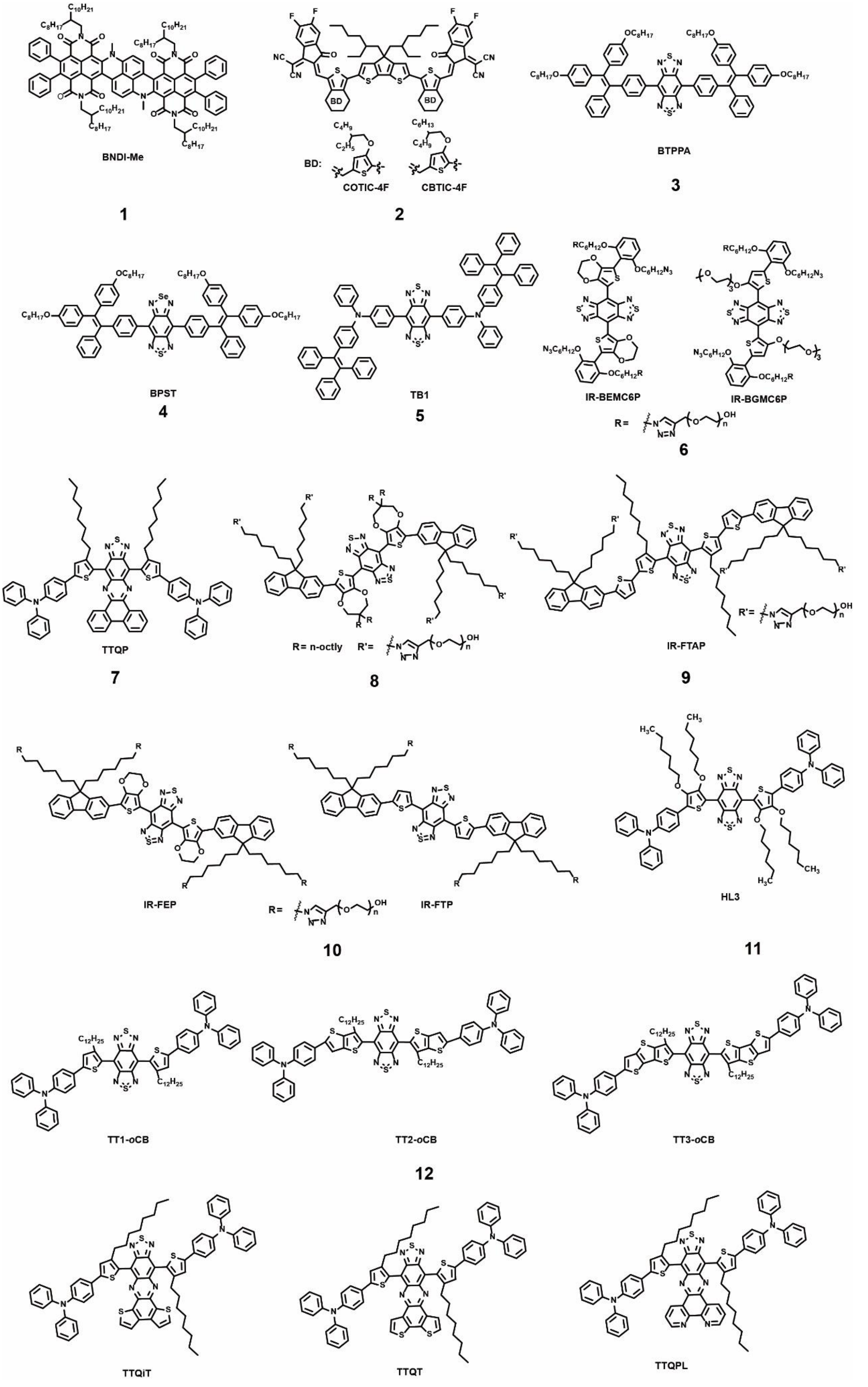
BNDI-Me
1
COTIC-4F
CBTIC-4F
2
BTPPA
3
BPST
4
TB1
5
IR-BEMC6P
IR-BGMC6P
6
TTQP
7
R= n-octly
8
IR-FTAP
9
IR-FEP
IR-FTP
10
HL3
11
TT1-oCB
TT2-oCB
TT3-oCB
12
TTQiT
TTQT
TTQPL

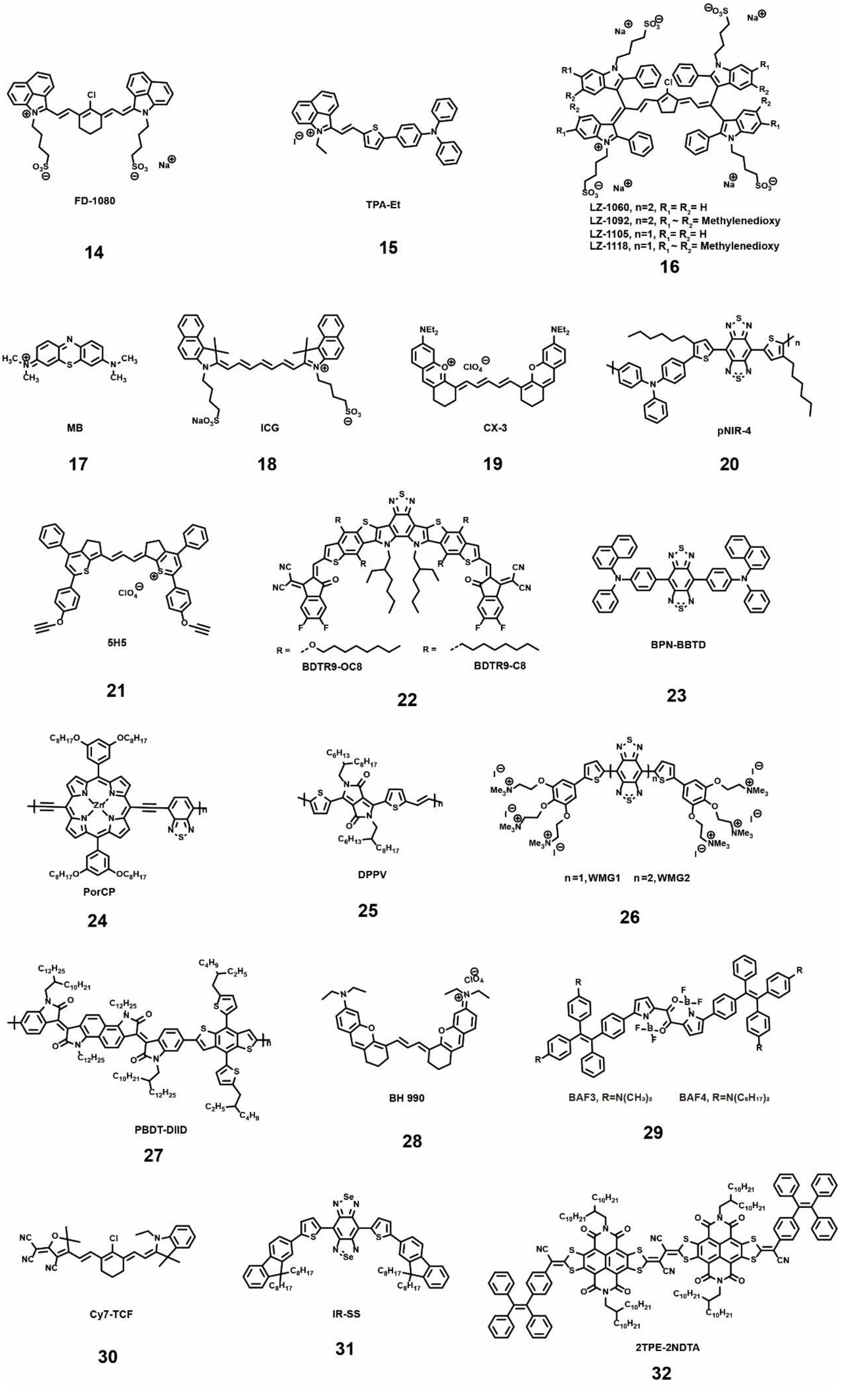
FD-1080
14
TPA-Et
15
LZ-1060, n=2, R1= R2= H
LZ-1092, n=2, R1 ~ R2= Methylenedioxy
LZ-1105, n=1, R1= R2= H
LZ-1118, n=1, R1 ~ R2= Methylenedioxy
16
MB
17
ICG
18
CX-3
19
pNIR-4
20
5H5
21
BDTR9-OC8
BDTR9-C8
22
BPN-BBTD
23
PorCP
24
DPPV
25
n=1,WMG1 n=2,WMG2
26
PBDT-DIID
27
BH 990
28
BAF3, R=N(CH3)2 BAF4, R=N(C8H17)2
29
Cy7-TCF
30
IR-SS
31
2TPE-2NDTA
32

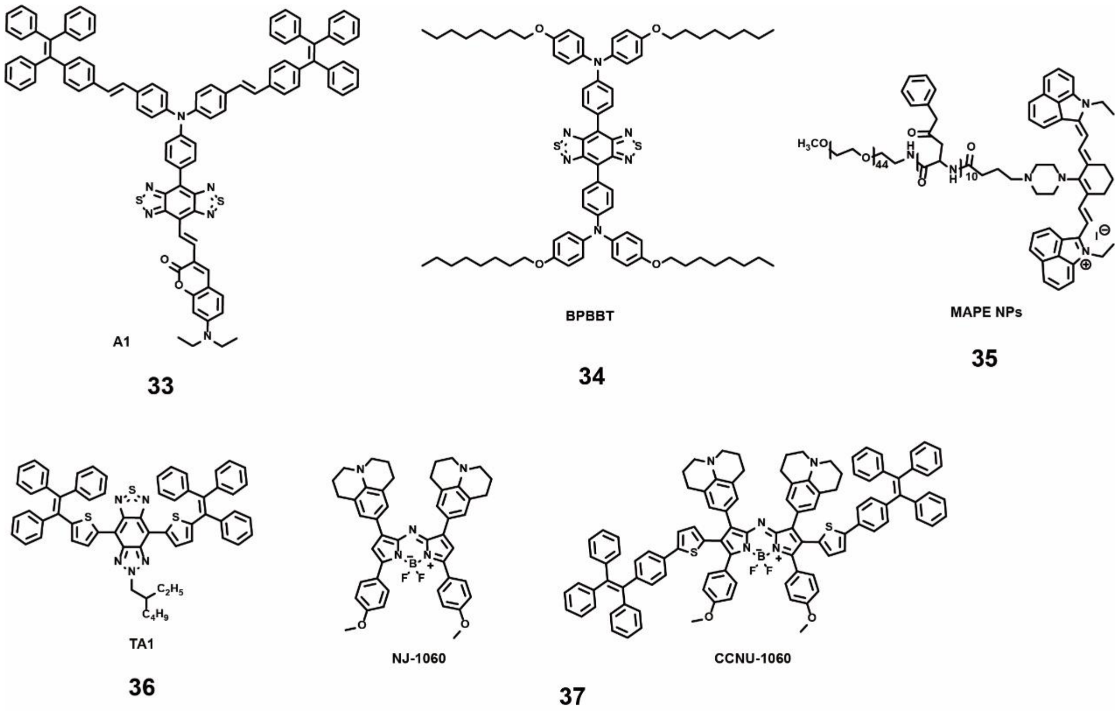

Figure S27. The chemical structure of organic molecules in Table S1.

Table S2. Fitting parameters of GSB at 868 nm for BNDI-H NPs and 854 nm for BNDI-Me NPs. The decays were fit with the following biexponetial function: I $=A_1e^{-t/\tau1}+A_2e^{-t/\tau2}$. A_1 and A_2 represent the fraction of excited population of the associated component. Average τ was calculated by formula: $\tau_1 \times A_1 + \tau_2 \times A_2$.

Materials	τ_1	A_1	τ_2	A_2	average τ
BNDI-H NPs	1.23 ps	28.8%	13.4 ps	71.2%	9.90 ps
BNDI-Me NPs	2.97 ps	45.4%	48.7 ps	54.6%	27.94 ps

References

[1] X. Zhu, C. Liu, Z. Hu, H. Liu, J. Wang, Y. Wang, X. Wang, R. Ma, X. Zhang, H. Sun, Y. Liang, *Nano Res.* **2020**, *13*, 2570.

[2] W. Wu, Y. Q. Yang, Y. Yang, Y. M. Yang, H. Wang, K. Y. Zhang, L. Guo, H. F. Ge, J. Liu, H. Feng, *Int. J. Nanomed.* **2019**, *14*, 3571.

[3] W. Wu, Y. Yang, Y. Yang, Y. Yang, K. Zhang, L. Guo, H. Ge, X. Chen, J. Liu, H. Feng, *Small* **2019**, *15*, 1805549.

[4] Z. Sheng, B. Guo, D. Hu, S. Xu, W. Wu, W. H. Liew, K. Yao, J. Jiang, C. Liu, H. Zheng, B. Liu, *Adv. Mater.* **2018**, *30*, 1800766.

[5] R. Tian, H. Ma, Q. Yang, H. Wan, S. Zhu, S. Chandra, H. Sun, D. O. Kiesewetter, G. Niu, Y. Liang, X. Chen, *Chem. Sci.* **2019**, *10*, 326.

[6] Y. Li, M. Zha, G. Yang, S. Wang, J.-S. Ni, K. Li, *Chem. – Eur. J.* **2021**, *27*, 13085.

[7] H. Ma, C. Liu, Z. Hu, P. Yu, X. Zhu, R. Ma, Z. Sun, C.-H. Zhang, H. Sun, S. Zhu, Y. Liang, *Chem. Mater.* **2020**, *32*, 2061.

[8] Q. Yang, Z. Hu, S. Zhu, R. Ma, H. Ma, Z. Ma, H. Wan, T. Zhu, Z. Jiang, W. Liu, L. Jiao, H. Sun, Y. Liang, H. Dai, *J. Am. Chem. Soc.* **2018**, *140*, 1715.

[9] Q. Yang, Z. Ma, H. Wang, B. Zhou, S. Zhu, Y. Zhong, J. Wang, H. Wan, A. Antaris, R. Ma, X. Zhang, J. Yang, X. Zhang, H. Sun, W. Liu, Y. Liang, H. Dai, *Adv. Mater.* **2017**, *29*, 1605497.

[10] Y. Xu, C. Li, R. Xu, N. Zhang, Z. Wang, X. Jing, Z. Yang, D. Dang, P. Zhang, L. Meng, *Chem. Sci.* **2020**, *11*, 8157.

[11] S. Liu, R. Chen, J. Zhang, Y. Li, M. He, X. Fan, H. Zhang, X. Lu, R. T. K. Kwok, H. Lin, J. W. Y. Lam, J. Qian, B. Z. Tang, *ACS Nano* **2020**, *14*, 14228.

[12] Y. Li, M. Zha, T. Kang, C. Li, X. Wu, S. Wang, S.-B. Lu, Y.-S. Lee, Y.-R. Wu, J.-S. Ni, K. Li, *Small* **2022**, *18*, 2105362.

[13] C. Sun, B. Li, M. Zhao, S. Wang, Z. Lei, L. Lu, H. Zhang, L. Feng, C. Dou, D. Yin, H. Xu, Y. Cheng, F. Zhang, *J. Am. Chem. Soc.* **2019**, *141*, 19221.

[14] C. Teng, S. Zhang, Y. Tian, Q. Cheng, H. Dang, D. Yin, L. Yan, *Nanomedicine (N. Y., NY, U. S.)* **2022**, *44*, 102574.

[15] B. Li, M. Zhao, L. Feng, C. Dou, S. Ding, G. Zhou, L. Lu, H. Zhang, F. Chen, X. Li, G. Li, S. Zhao, C. Jiang, Y. Wang, D. Zhao, Y. Cheng, F. Zhang, *Nat. Commun.* **2020**, *11*, 3102.

[16] D. Xue, D. Wu, Z. Lu, J. Neuhaus, A. Zebibula, Z. Feng, S. Cheng, J. Zhou, J. Qian, G. Li, *Engineering* **2022**, DOI: 10.1016/j.eng.2021.07.032.

[17] H.-J. Zhou, T.-B. Ren, *Chem. – Asian J.* **2022**, *17*, e202200147.

[18] Z. Lei, C. Sun, P. Pei, S. Wang, D. Li, X. Zhang, F. Zhang, *Angew. Chem. Int. Ed.* **2019**, *58*, 8166.

[19] S. Liu, H. Ou, Y. Li, H. Zhang, J. Liu, X. Lu, R. T. K. Kwok, J. W. Y. Lam, D. Ding, B. Z. Tang, *J. Am. Chem. Soc.* **2020**, *142*, 15146.

[20] B. Ding, Y. Xiao, H. Zhou, X. Zhang, C. Qu, F. Xu, Z. Deng, Z. Cheng, X. Hong, *J. Med. Chem.* **2019**, *62*, 2049.

[21] Y. Zhu, H. Lai, H. Guo, D. Peng, L. Han, Y. Gu, Z. Wei, D. Zhao, N. Zheng, D. Hu, L. Xi, F. He, L. Tian, *Angew. Chem. Int. Ed.* **2022**, *61*, e202117433.

[22] N. Alifu, A. Zebibula, J. Qi, H. Zhang, C. Sun, X. Yu, D. Xue, J. W. Y. Lam, G. Li, J. Qian, B. Z. Tang, *ACS Nano* **2018**, *12*, 11282.

[23] B. Guo, G. Feng, P. N. Manghnani, X. Cai, J. Liu, W. Wu, S. Xu, X. Cheng, C. Teh, B. Liu, *Small* **2016**, *12*, 6243.

[24] Y. Lyu, J. Zeng, Y. Jiang, X. Zhen, T. Wang, S. Qiu, X. Lou, M. Gao, K. Pu, *ACS Nano* **2018**, *12*, 1801.

[25] B. Wang, G. Feng, M. Seifrid, M. Wang, B. Liu, G. C. Bazan, *Angew. Chem. Int. Ed.* **2017**, *56*, 16063.

[26] X. Duan, Q. Zhang, Y. Jiang, X. Wu, X. Yue, Y. Geng, J. Shen, D. Ding, *Adv. Mater.* **2022**, *34*, 2200179.

[27] H. Bian, D. Ma, X. Zhang, K. Xin, Y. Yang, X. Peng, Y. Xiao, *Small* **2021**, *17*, 2100398.

[28] Z. Jiang, C. Zhang, X. Wang, M. Yan, Z. Ling, Y. Chen, Z. Liu, *Angew. Chem. Int. Ed.* **2021**, *60*, 22376.

[29] X. Mu, Y. Lu, F. Wu, Y. Wei, H. Ma, Y. Zhao, J. Sun, S. Liu, X. Zhou, Z. Li, *Adv. Mater.* **2020**, *32*, 1906711.

[30] S. Li, Q. Deng, Y. Zhang, X. Li, G. Wen, X. Cui, Y. Wan, Y. Huang, J. Chen, Z. Liu, L. Wang, C.-S. Lee, *Adv. Mater.* **2020**, *32*, 2001146.

[31] Z. Zhao, C. Chen, W. Wu, F. Wang, L. Du, X. Zhang, Y. Xiong, X. He, Y. Cai, R. T. K. Kwok, J. W. Y. Lam, X. Gao, P. Sun, D. L. Phillips, D. Ding, B. Z. Tang, *Nat. Commun.* **2019**, *10*, 768.

[32] Q. Qu, Z. Zhang, X. Guo, J. Yang, C. Cao, C. Li, H. Zhang, P. Xu, Z. Hu, J. Tian, *J. Nanobiotechnol.* **2022**, *20*, 143.

[33] S. Gao, G. Wei, S. Zhang, B. Zheng, J. Xu, G. Chen, M. Li, S. Song, W. Fu, Z. Xiao, W. Lu, *Nat. Commun.* **2019**, *10*, 2206.

[34] Q. Cheng, Y. Tian, H. Dang, C. Teng, K. Xie, D. Yin, L. Yan, *Adv. healthcare mater.* **2022**, *11*, 2101697.

[35]B. Guo, Z. Huang, Q. Shi, E. Middha, S. Xu, L. Li, M. Wu, J. Jiang, Q. Hu, Z. Fu, B. Liu, *Adv. Funct. Mater.* **2020**, *30*, 1907093.

[36] W. Huang, H. Yang, Z. Hu, Y. Fan, X. Guan, W. Feng, Z. Liu, Y. Sun, *Adv. healthcare mater.* **2021**, *10*, 2101003.
